# AI-Driven Robotics Laboratory Identifies Pharmacological TNIK Inhibition as a Potent Senomorphic Agent

**DOI:** 10.14336/AD.2024.1492

**Published:** 2026-02-13

**Authors:** Qiuqiong Tang, Deyong Xiao, Alexander Veviorskiy, Ying Xin, Sarah W.Y. Lok, Fadi E. Pulous, Peiran Zhang, Yunfeng Zhu, Yongming Ma, Xiao Hu, Shoulai Gu, Chenting Zong, Sabina Mukba, Mikhail Korzinkin, Frank W. Pun, Man Zhang, Alex Aliper, Lijuan Wu, Feng Ren, Li Zhang, Alex Zhavoronkov

**Affiliations:** ^1^Insilico Medicine US Inc, Cambridge, MA 02138, USA.; ^2^Insilico Medicine Suzhou Ltd., Suzhou, Jiangsu, China.; ^3^Insilico Medicine AI Limited, Masdar, Abu Dhabi, UAE.; ^4^Insilico Medicine Hong Kong Ltd., Hong Kong, China.; ^5^Insilico Medicine Shanghai Ltd., Shanghai, China.; ^6^Department of Dermatology, the affiliated Suzhou Hospital of Nanjing Medical University, Suzhou, Jiangsu, China.; ^7^Department of Geriatric medicine, the affiliated Suzhou Hospital of Nanjing Medical University, Suzhou, Jiangsu, China.; ^8^Buck Institute for Research on Aging, Novato, CA 94945, USA

**Keywords:** TNIK inhibition, Cellular senescence, Extracellular matrix, SASP, Senomorphic, Robotics drug discovery

## Abstract

Assessing impact on the hallmarks of aging has emerged as a novel method for prioritizing dual-purpose longevity therapeutic targets and developing drugs simultaneously targeting aging and disease. Cellular senescence, a central hallmark of aging, progressively induces cellular growth arrest and accelerates the production of a pro-inflammatory senescence-associated secretory phenotype (SASP). TGF-β signaling is situated at the center of multiple senescence-associated and aging-associated signaling pathways, and its inhibition may be favorable for aging-related disorders. A recently developed Traf2- and Nck-interacting kinase (TNIK) inhibitor, INS018_055, was identified as a potent, novel anti-fibrotic agent affecting multiple hallmarks of aging across fibrotic diseases. Thus, we hypothesized that TNIK is a potential senescence modulator and INS018_055 could attenuate senescent cell accumulation to treat specific age-related pathological processes. Using a fully automated robotics laboratory designed for automated, highly parallel, and iterative phenotypic and multi-omic analyses, we determined that pharmacological or siRNA-mediated TNIK inhibition decreased cellular senescence in multiple experimental senescence models. INS018_055 mechanistically demonstrated senomorphic activity through its reduction of SASP. Furthermore, transcriptomics analysis revealed that INS018_055 treatment reduced aging signatures and extracellular matrix fibronectin through TGF-β signaling. These findings reveal TNIK’s previously unappreciated role in cellular senescence and INS018_055’s senomorphic potential in mitigating processes well-established as driving organismal aging. Thus, TNIK inhibition as a novel senomorphic strategy may inform future therapeutic approaches for diverse aging-related diseases.

## INTRODUCTION

Aging is a significant risk factor for many chronic diseases, driving substantial morbidity and excess healthcare expenditures [1]. In the last decade, cellular and molecular hallmarks of aging that collectively drive the aging process have been identified and categorized [2, 3]. Therapeutic modulation of these pathways has been shown to slow the biological aging cascade and potentially delay the onset of several age-related diseases [4]. Cellular senescence, defined as the stable arrest of the cell cycle in young and aged individuals, is one of the major hallmarks implicated in the exhaustion of cellular reparative capabilities [5]. During senescence, cells acquire a senescence-associated secretory phenotype (SASP), characterized by the secretion of pro-inflammatory cytokines and tumor-promoting growth factors impairing several physiological functions [4]. Pharmacological agents termed senotherapeutics have shown preclinical success in targeting senescent cells and can be classified into two categories: i. senolytics induce programmed cell death in senescent cells; ii. senomorphics suppress the pro-inflammatory SASP (“senostasis”) [6, 7]. Notably, senescence is beneficial in limiting damaged cell expansion and negative consequences of age-related pathologies [8]. Thus, the need for novel senotherapeutic interventions is context-specific and must mitigate the accumulation of senescent cells specifically in settings associated with tissue deterioration that drives age-related pathologies [4].

One such pathological setting potentially susceptible to senotherapeutic treatment is idiopathic pulmonary fibrosis (IPF), an age-related, degenerative lung disease with no approved curative treatment available. Cellular senescence has been shown to be pivotal in IPF pathogenesis, driving pro-aging stressors involving telomere attrition, oxidative stress, DNA damage, and proteome instability [9]. Senotherapeutics have been proposed as treatments for IPF and other age-related pulmonary diseases [9-11], with a number of senolytics having reached human clinical trials in IPF [12, 13].

A critical driver of recent progress in senotherapeutic research is the significant computational advances of the last two decades, particularly in the field of artificial intelligence (AI)-driven drug discovery [14-18]. The newly identified senotherapeutic targets and novel inhibitors discovered through AI-based methods have opened the door for therapies that directly act upon the aging process. Anti-aging interventions termed “geroprotectors” [19, 20] have received growing attention in the last decade particularly with the preclinical successes of mammalian target of rapamycin (mTOR) inhibitors like metformin and rapamycin [21]. Although metformin has been approved for the treatment of type 2 diabetes, the clinical translation of these drugs for the treatment of biological aging has stalled amidst considerable controversy. Thus, new computational approaches driven by the advent of generative AI have focused on identifying new targets that may be useful for extending longevity while also acting upon biological processes that drive many age-related disorders. AI-driven discovery of aging-related biomarkers or “aging clocks'' has yielded several promising targets for the development of potential longevity therapeutics that are currently being tested and validated in multiple, independent laboratories [22-26].

One such dual-purpose aging and aging-related disease target is Traf2- and Nck-interacting kinase (TNIK), a member of the germinal center kinase (GCK) family. TNIK plays essential roles in multiple hallmarks of aging, making it an intriguing senotherapeutic target [27], with recent studies implicating TNIK in multiple age-related diseases [27, 28]. For example, TNIK has been shown to regulate TGF-β signaling in fibroblasts to promote proliferation and differentiation into myofibroblasts while enhancing cell survival by activating c-Jun N-terminus kinase (JNK) [29], and TNIK inhibition suppresses tumor cell growth and invasion in colon and ovarian cancer through Wnt signaling [30, 31].

We recently identified TNIK as a crucial pro-fibrotic factor and developed INS018_055 (Rentosertib), a selective, potent TNIK inhibitor, which exhibited strong anti-fibrotic effects in murine IPF, skin fibrosis, and kidney fibrosis models [32]. INS018_055 attenuated fibrosis by reducing extracellular matrix (ECM) deposition, epithelial-to-mesenchymal (EMT), and fibroblast-to-myofibroblast (FMT) pro-fibrotic cellular programs. In addition to the strong inhibition of TNIK, INS018_055 was designed to mildly inhibit WNT, Yes, TGF-β, and several other pathways implicated in aging, fibrosis, and senescence [32]. We thus hypothesized that INS018_055 might offer senotherapeutic benefit for combating cellular senescence in age-related fibrosis by inhibiting TNIK and other targets and pathways implicated in fibrosis and aging.

**Figure 1.**
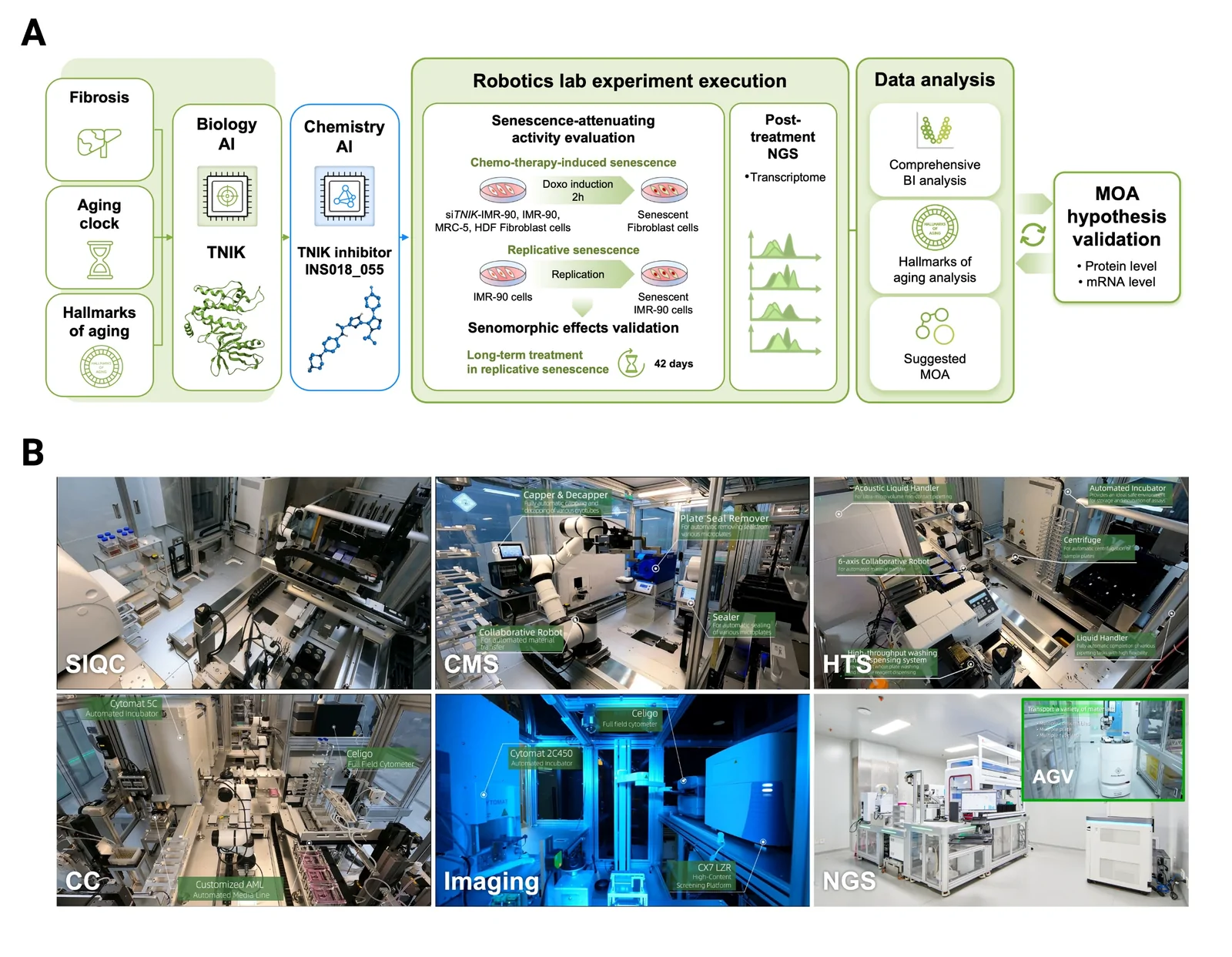
**AI-powered aging research platform and robotics lab-powered workflow**. (**A**) Simplified schematic of project workflow for this study. (**B**) Layouts of six functional modules of the robotics lab: BI, bioinformatics; NGS, next-generation sequencing; MOA, mode of action; SIQC, sample intake, and quality control; CC, cell culture; CMS, compound management system; HTS, high-throughput screening; NGS, next-generation sequencing. AGV, autonomous guided vehicle

To study drivers of aging and disease and to train and test machine learning models we refer to as Life Models [16, 33, 34], we developed a fully automated robotics facility allowing for generation of full-genome sequencing, methylation, expression, and phenotypic data from each individual sample. AI models trained on the multi-omic data generated allow for prediction of therapeutic targets and evaluation of phenotypic response to treatment with AI-generated or -identified compounds [35]. Here, we explored the senescence-attenuating properties of the AI-generated TNIK inhibitor INS018_055 in multiple senescence cellular models using the automated robotics system (Fig. 1 and Supplementary video 1). The robotics lab integrates six distinct functional modules: Sample Intake and Quality Control (SIQC), Compound Management System (CMS), High-Throughput Screening (HTS), Cell Culture (CC), High-Content Imaging (IMG), and Next-Generation Sequencing (NGS). The lab aims to integrate target identification, validation, drug discovery, and translational research seamlessly to expedite AI-driven drug discovery processes. In this study, we utilized specific functional modules, singularly and in combination, to evaluate INS018_055’s senotherapeutic properties. INS018_055 demonstrated a senescence-attenuating effect by reducing ECM remodeling and SASP proinflammatory cytokine production. Mechanistically, INS018_055 altered ECM stiffness and SASP by inhibiting TGF-β and Wnt-signaling mediated transcriptional activity. These novel findings suggest the vital role of TNIK in cellular senescence and the potential of INS018_055 as a senomorphic molecule, providing a promising therapeutic approach for treating age-related diseases.

## MATERIALS AND METHODS

### Robotics lab experiment execution

The present study was conducted in the AI-powered sixth-generation robotics laboratory according to the workflow illustrated in Supplementary Figure 1. Briefly, cellular samples are processed and quality-checked within the Sample Input and Quality Control (SIQC) module. These standardized samples undergo further analysis in the Next-Generation Sequencing (NGS) module, assaying DNA, RNA, and methylation to generate machine-readable data. This pre-processed multi-omics data is directed into the AI-driven target discovery platform PandaOmics [36]. In parallel with these target or indication predictions, the cell culture (CC) model facilitates maintenance of cell cultures. Upon receiving predictive data and corresponding compounds from the AI platform, the robotics laboratory embarks on wet lab validation, deploying siRNA and/or pharmacologic inhibition within the CC and/or High-throughput Screening (HTS) modules. Post-treatment effects are evaluated with diverse metrics, including cellular vitality, morphology, and variations in gene expression. The concluding step involves near real-time reinforcement learning derived from prediction and validation tests, either rewarding or penalizing the AI model based on experimental outcomes, establishing a feedback loop that increasingly refines the AI's proficiency in target discovery and indication prediction.

The potential senescence-attenuating activity of INS018_055 was first evaluated in chemotherapy-induced senescence and replicative senescence models, which engaged the CMS, CC, and HTS modules. Next, post-treatment cellular samples were collected at multiple time points for RNA sequencing analysis in the NGS module, followed by SA-β-gal staining in the high-content image module (Fig. 1B). The post-treatment transcriptome data were then entered into our comprehensive data analysis pipeline, which included comprehensive bioinformatic differential expression analysis, hallmarks of aging analysis, and mechanisms of action (MOA) analysis. To confirm these findings, further wet lab validation experiments were designed and conducted to elucidate INS018_055’s mechanism of action.

### Cell culture

Normal human lung fibroblast cell lines IMR-90 and MRC-5 were obtained from ATCC and cultured in MEM medium (Procell, cat# PM150411) supplemented with 10% fetal bovine serum (Gibco, cat# A5669701), 1% Non-Essential Amino Acids (Gibco, cat# 11140050), 1mM Sodium Pyruvate (Gibco, cat# 11360070) and 1% penicillin-streptomycin (P/S, Gibco, cat# 15140122). Primary human dermal fibroblast (HDF) from healthy donors were purchased from Lonza (cat# CC-2511, batch # 22TL346467"). and cultured in FGM™-2 Fibroblast Growth Medium-2 BulletKit™ (cat# CC-3132). The cell line was routinely tested for mycoplasma contamination (Lonza, cat# LT07-710) and authenticated with short tandem repeat (STR) assays.

### Materials

TNIK inhibitor INS018_055 was supplied by Insilico Medicine internally. Other reference compounds were purchased from Medchemexpress (MCE), including 1) the senolytic compound ABT-263 (MCE, cat# HY-10087), and 2) the senomorphic nordihydroguaiaretic acid (NDGA, MCE, cat# HY-N0198) targeting HSP90, rapamycin (MCE, cat# HY-10219), torin 1 (MCE, cat# HY-13003) and metformin (MCE, cat# HY-17471A). All compounds were dissolved in dimethyl sulfoxide (DMSO, Solarbio, cat# D8371).

### Chemotherapy-induced cellular senescence model

IMR-90, MRC-5, or primary HDF cells were grown in T75 cell culture flasks at cell confluency of <80% and cell viability at > 90% for senescence model establishment. Cells at passage 10-11 were used for the following experiments. Cells were seeded into 96-well plates at a volume of 100μL per well and incubated at 37°C overnight. Doxorubicin was diluted to 625 nM using a complete cell medium with Dulbecco’s phosphate buffer saline (ThermoFisher, cat# 14190144) as a blank control. Culture medium was exchanged with 100μL per well of medium containing doxorubicin, and plates were incubated at 37°C for 2 hours. Following incubation, the complete medium was replaced with respective compounds, and treatment continued for 72 hours. After 72 hours, the SA-β-gal staining and RT-qPCR were then performed.

### Replicative senescence model induction

Replicative senescence was performed by cell passaging with IMR-90 cells. Cells were passaged to senescence and the population doubling level was increased by amplifying the culture system. IMR-90 cells were sub-cultured at 1×10^6^ cells/T175 flask to shorten the analysis time. Cells at different passages (P12, P15, P16, P17, P18) were collected for SA-β-gal, RT-qPCR, and RNA sequencing. For SA-β-gal and RT-qPCR, the compound treatment duration was 72 hours. For transcriptomic analysis, the compound treatment duration was continued during cell passaging, and cellular samples were collected at multiple time points.

### Senescence-associated beta-galactosidase (SA-β-gal) staining

After compound treatment, SA-β-gal staining was performed using the SA-β-gal staining kit according to the manufacturer’s protocol (Beyotime, cat# C0602). Briefly, cells were fixed using 100μL fixation buffer and incubated for 15 mins at room temperature (RT), then washed twice with 100μL PBS for 3 mins at RT and final aspiration. Subsequently, 100μL of staining solution was added to each well; plates were incubated overnight in a CO_2_-free incubator at 37°C. The next day, the staining solution was aspirated, and cells were washed with 100μL PBS. Then, 100μL of 4μg/mL Hoechst solution was added to each well, incubated for 15 mins at RT, and washed twice with PBS. The plates were sealed and scanned using ArrayScan High-Content Screening System (Thermo Fisher Scientific, CX7 LZR) in 5 channels (DAPI: Ex405/Em446/37; SA-β-gal: 590(Amber)-Brightfield, 617 (Red)-Brightfield, 447(Blue)-Brightfield, 530(Green)-Brightfield) with 25 fields per well and 10×magnification (binning 2×2, 1104×1104). The size of each field is 885.54 × 885.54 μm.

### siRNA knock-down

IMR-90 cell suspensions were seeded in 96-well plates at a final cell number of 8000 per well and transfected two siRNAs for TNIK gene and non-targeting control (siNC) with RNAi duplex-Lipofectamine™ RNAiMAX Kit (ThermoFisher, cat 13778150) for 72 h post-transfection at 37°C, 5 % CO_2_. Three replicates per cell line were performed. All siRNAs were purchased from GENScript and used at a final concentration of 10 nM. siRNA sequences are listed in the following table (Table 1).

**Table 1:** The siRNA sequences.

siRNA	Sense strand	Antisense strand
NC-siRNA	AGUAGCGUACGAGGAGUGCUU	AGUAGCGUACGAGGAGUGCUU
TNIK-siRNA-1	UUUUAAAUUAGAAAUAACGCC	CGUUAUUUCUAAUUUAAAAGA
TNIK-siRNA-2	UCUUUGUUCUAUCAAUAUGGU	CAUAUUGAUAGAACAAAGAAG

### Quantitative real-time polymerase chain reaction (RT-qPCR) analysis

Total RNA extraction was performed using FastPure Cell/Tissue Total RNA Isolation Kit (Vazyme, cat# RC112-01), and complementary DNA (cDNA) was synthesized after reverse transcription of RNA using HiScript III 1st Strand cDNA Synthesis Kit (Vazyme, cat# R312-01) according to the manufacturer’s protocol. Quantitative RT-PCRs were performed in triplicate using ChamQ SYBR qPCR Master Mix (Vazyme, cat# Q321-03). The primers used are listed in the Table 2. Samples were run on LightCycler® 480 real-time PCR Instrument (Roche) with the following program: pre-denaturation (30s, 95°C), PCR amplification (40 cycles of denaturing at 95°C for 10s and annealing/extension at 60°C for 30s). LightCycler® 480 software was used to compute CT values. All values were normalized to those of β-actin.

### RNA sequencing sample preparation

RNA was isolated using the Magnetic Tissue/Cell/Blood Total RNA Kit (TIANGEN, cat# DP761) following the manufacturer’s instructions. Total RNA samples were evaluated for integrity using an Agilent 5400 Bioanalyzer. RNA-seq libraries were constructed using the KAPA mRNA HyperPrep Kit (Roche, cat# KR1352) and sequenced on the Illumina NovaSeq NovaSeq6000 platform in the 150 nt, paired-end configuration.

### RNA sequencing analysis

*RNA-seq data integration in PandaOmics:* RNA sequencing data was processed using hardware and software tools from Illumina. The “BCL Convert” tool was used to convert BCL files to FASTQ files, while the “DRAGEN RNA Pipeline” was used to extract raw gene expression counts from FASTQ files. Next, raw gene expression counts were uploaded in PandaOmics [36] and pre-processed according to the PandaOmics pipeline that automatically defines data type (raw counts, normalized gene expression values, or log-transformed gene expression values), recommends normalization method for further analysis and filters out non-protein coding genes. Upper quantile normalization and log2-transformation were applied for the gene expression matrix. After that, comparisons between DMSO and INS018_055 treated groups between all passages were created, and differential expression analysis for all analyzed comparisons was performed using the Limma package. Obtained gene-wise p-values were corrected for multiple tests using the Benjamini-Hochberg procedure to control the false discovery rate (FDR).

**Table 2:** The list of primers.

Primers	Sequence
CDKN2A (P16) forward primer	CTCGTGCTGATGCTACTGAGGA
CDKN2A (P16) reverse primer	GGTCGGCGCAGTTGGGCTCC
CDKN1A (P21) forward primer	AGGTGGACCTGGAGACTCTCAG
CDKN1A (P21) forward primer	TCCTCTTGGAGAAGATCAGCCG
IL6 forward primer	AGACAGCCACTCACCTCTTCAG
IL6 reverse primer	TTCTGCCAGTGCCTCTTTGCTG
IL8 forward primer	ACTGAGAGTGATTGAGAGTGGA
IL8 reverse primer	TGAATTCTCAGCCCTCTTCAAA
TGFB1 forward primer	TACCTGAACCCGTGTTGCTCTC
TGFB1 reverse primer	GTTGCTGAGGTATCGCCAGGAA
IL1B forward primer	CCACAGACCTTCCAGGAGAATG
IL1B reverse primer	GTGCAGTTCAGTGATCGTACAGG
TNIK forward primer	ACAGTGGCTGTCAGCGACATAC
TNIK reverse primer	ATACTGCCGCTGAAACTGTCCG
β-actin forward primer	CACCATTGGCAATGAGCGGTTC
β-actin reverse primer	AGGTCTTTGCGGATGTCCACGT

*Regression analysis:* Upper quantile normalized RNA-seq data was downloaded from PandaOmics and used for the regression experiments. Regression analysis was conducted independently for DMSO and INS018_055 samples. Expression values between the passages were averaged and regression analysis was performed using statsmodels.regression.linear_model. OLS python package.

*Gene Set Enrichment Analysis and Transcription Factor enrichment analysis:* Gene Set Enrichment Analysis (GSEA) was performed to compare DMSO 18 passage vs. DMSO 12 passage, INS018_055 18 passages vs. DMSO 18 passage, and INS018_055 18 passages vs. DMSO 12 passage. Differential expression analysis results for these comparisons were used as input for GSEA analysis. Each gene was ranked according to the value derived from the multiplication of LogFoldChanges and -log10(p-value). Then, gseapy.prerank python function was used for the analysis and visualisation. Transcription Factors (TFs) enrichment analysis was performed using the gseapy package with the enrichr() function according to standard protocols. The primary library “ARCHS4_Coexpression” from the ChEA3 database was selected as the gene set for transcription factor enrichment analysis. TFs enrichment results were visualized on a bar plot using the matplotlib.pyplot Python package. Each bar on the plot corresponds to a significantly perturbed gene regulated by the corresponding TF. Significantly (FDR < 0.05) up-regulated and down-regulated genes are colored red and blue, respectively.

*Expression changes in hallmarks of aging:* Analysis of gene expression changes in hallmarks of aging was performed using upper quantile normalized RNA-seq data downloaded from PandaOmics. Then, z-score normalization between samples was applied to the expression matrix. Z-normalized expression changes of aging hallmarks were visualized on a heatmap using clustermap () function from the seaborn python package with turned “col_cluster=True” parameter.

### Western blotting assay

Cell proteins were extracted using RIPA lysis buffer (Beyotime, cat# P0013B, China) supplemented with 1% protease inhibitor cocktail tablets (Beyotime, cat# P1005, China). Protein lysate concentration was quantified using PIERCE BCA PROTEIN ASSAY KIT (Invitrogen, cat#23225, USA). The proteins were separated by SDS-PAGE on 4-20% gel (Genscript, cat# M42012C) and transferred to nitrocellulose membranes (Invitrogen, cat# IB23001). The membranes were blocked with 3% BSA (Beyotime, cat# ST023-200G) and were incubated with primary antibody overnight at 4 °C. The membranes were incubated with secondary antibodies before observing the targets with the Azure Biosystems C300 gel documentation system. Β-actin was used as an internal reference (Table 3).

**Table 3:** List of antibodies used in this study.

Antibody	Company	Cat#	Dilution
**TNIK antibody**	Cell Signaling Technology	32712S	1:1000
**Phospho-TNIK (Ser764) antibody**	Bioss	BS5598R	1:1000
**SMAD2/3 antibody**	Cell Signaling Technology	3102	1:1000
**phospho-SMAD2/3 antibody**	Cell Signaling Technology	8828	1:1000
**Fibronectin antibody**	Abcam	Ab2413	1:1000
**Beta-actin antibody**	Cell Signaling Technology	3700T	1:1000
**Anti-rabbit IgG, HRP-linked antibody**	Cell Signaling Technology	7074P2	1:5000
**MMP-2 antibody**	Cell Signaling Technology	87809	1:1000

### Statistical analysis

All *in vitro* experiments were performed in triplicates. GraphPad Prism 10.1.0 was used to collect and analyze data. For n>6, statistical significance was determined by unpaired two-tailed Student’s t-test. For n<6 or values are not normally distributed, statistical significance was determined by non-parametric tests including the Mann-Whitney test and Kruskal-Wallis test. Detailed information of individual tests is shown in corresponding figure legends. A p-value <0.05 was considered statistically significant for all statistical tests. ^*^p < 0.05; ^**^ p < 0.01. For the analysis of effect size, Cohen’s d was used, and the d value was presented in the supplementary Table 1. In unpaired two-tailed Student's t-test, mean, standard deviation (SD), and n number were used. In non-parametric tests, Mann-Whitney-U or Kruskal-Wallis-H was used for Cohen’s d-value calculation.

## RESULTS

### TNIK inhibition impairs chemotherapy-induced cellular senescence

**Figure 2.**
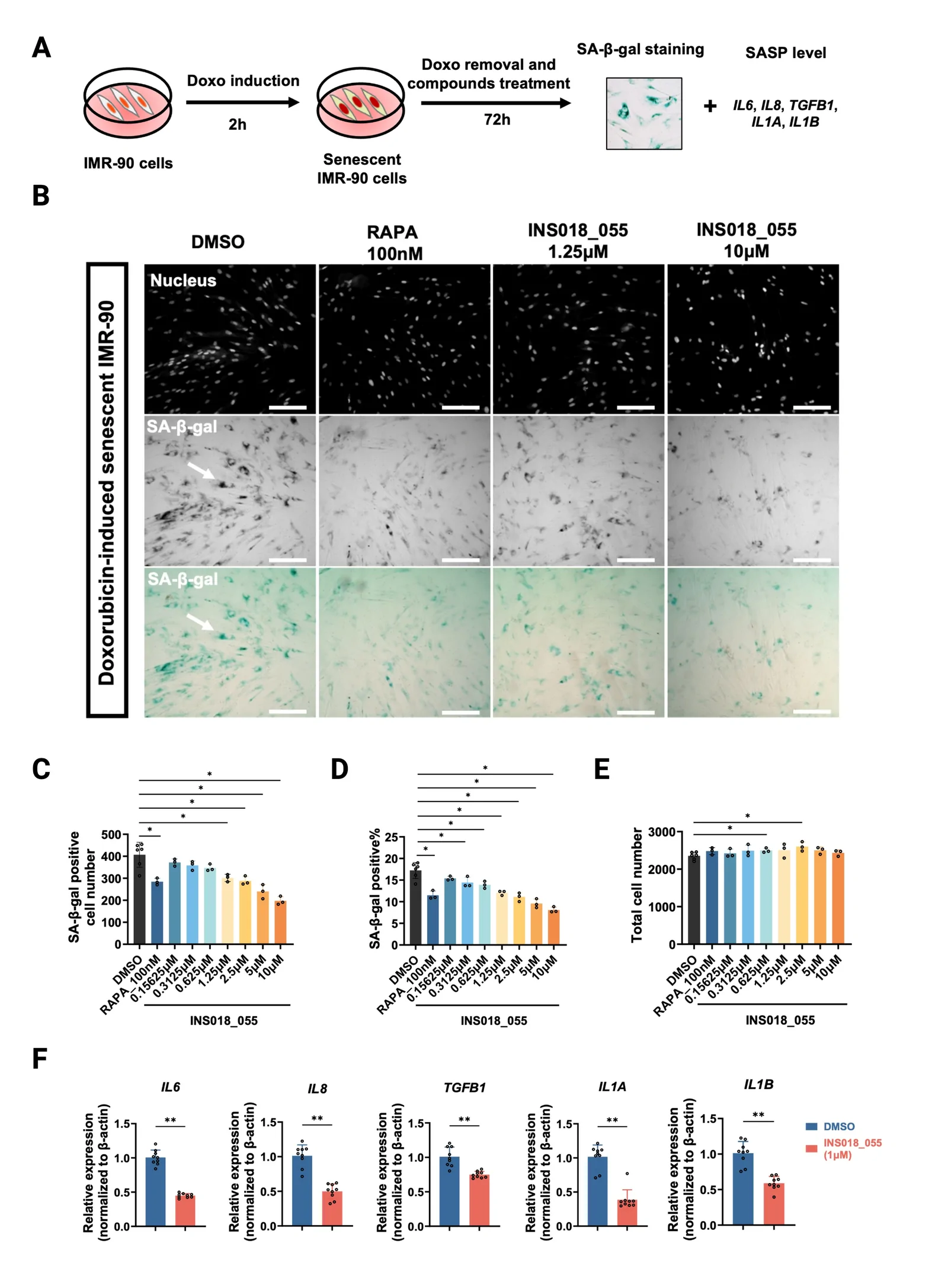
**Identification and testing of INS018_055 senomorphic activity in the chemotherapy-induced IMR-90 senescence model**. (**A**) Schematic of the chemotherapy-induced IMR-90 senescence model. IMR-90 cells were treated with doxorubicin for 2 hours to induce cellular senescence, and cells were then treated with INS018_055 and respective controls for an additional 72 hours. Cellular senescence was evaluated by the detection of SA-β-gal and SASP factor expression. (**B**) Representative staining images of doxorubicin-induced senescent IMR-90 cells treated with DMSO, 100nM rapamycin (RAPA), 1.25 and 10µM INS018_055. Top Row: Hoechst middle row: grey-scale SA-β-gal signal, bottom row: bright field signal. White arrows are SA-β-gal positive cells. Scale bar = 200 μm. (**C**) SA-β-gal positive cell number quantitation in samples treated with DMSO, RAPA, and INS018_055 at indicated concentrations. DMSO group: n=6; Other groups: n=3; ^*^p < 0.05; Mann-Whitney test. (**D**) Percentage of SA-β-gal positive cells in different groups. DMSO group: n=6; Other groups: n=3; ^*^p < 0.05; Mann-Whitney test. (**E**) Total cell number quantitation from groups analyzed in (C-D). DMSO group: n=6; Other groups: n=3; Mann-Whitney test. (**F**) Expression analysis of cell cycle regulators and SASP detected by RT-qPCR. Relative expression of *IL6*, *IL8*, *TGFB1*, *IL1A*, and *IL1B* were normalized against β-actin. n=9 per group; ^*^p < 0.05; ^**^p < 0.01; unpaired two-tailed Student's t-test.

To evaluate the senotherapeutic potential of INS018_055, we generated a chemically induced cellular senescence model using doxorubicin treatment on IMR-90 lung fibroblasts (Fig. 2A). In parallel, we developed a fully automated high-content imaging analysis pipeline for SA-β-gal staining by which thousands of cells in individual wells could be classified in an unbiased way into the SA-β-gal-positive (senescent) or -negative (non-senescent). With this robust and accurate methodology for senotherapeutic screening and validation, we observed increased SA-β-gal staining confirming cellular senescence upon 2-hour doxorubicin incubation (Supplementary Fig. 2A-C). To further validate this senescence model and the screen’s reproducibility, we examined the effects of known senolytics such as ABT-263 and two senomorphic mTOR inhibitors, rapamycin and torin 1. These compounds induced a substantial decrease in SA-β-gal-positive cells after 72 hours treatment (Supplementary Fig. 2A-C). ABT-263 treatment decreased the overall cell count (Supplementary Fig. 2A), whereas rapamycin and torin 1 reduced both SA-β-gal-positive cell numbers and the proportion of senescent cells (Supplementary Fig. 2B-C) without altering the total cell number (Supplementary Figure 2D). These findings indicate that the chemotherapy-induced cellular senescence model *in vitro* and the high-content imaging screening pipeline are sufficiently sensitive and robust for screening and validation of senotherapeutics, including senomorphics and senolytics.

Subsequently, we assessed INS018_055 and other senotherapeutic control compounds using this chemotherapy-induced cellular senescence model together with the high-content imaging-based pipeline. Intriguingly, INS018_055 treatment led to a significant reduction in senescence evidenced by a concentration-dependent decrease in the number and relative percentage of SA-β-gal-positive cells without decreasing total cell counts (Fig. 2B-E), suggesting a senomorphic effect. INS018_055’s senomorphic effect with unchanged total cell number was comparable to 100 nM rapamycin, highlighting INS018_055’s ability to reverse drug-induced cellular senescence. Additionally, IMR-90 cell toxicity (>20%) was not observed following INS018_055 treatment at any concentration (Supplementary Fig. 2E). Next, we performed qPCR analysis of SASP markers *IL6*, *IL8*, *TGFB*, *IL1A*, and *IL1B* to understand the phenotypic and signaling basis of INS018_055’s anti-senescence effect. Treatment with INS018_055 (1μM) significantly decreased the expression of TGF-β, IL6, IL8, IL1A, and IL1B compared to DMSO-treated controls following doxorubicin induction (Fig. 2F).

Next, we validated the anti-senescence activity of INS018_055 using the doxorubicin-induced MRC-5 cellular senescence model. Treatment with INS018_055 significantly reduced SA-β-gal-positive cell numbers and the proportion of senescent cells with only modest reduction in cell counts (Supplementary Fig. 3A-C), in agreement with findings from the IMR-90 senescent model. We also confirmed the senomorphic effects of INS018_055 in doxorubicin-induced primary human dermal fibroblast (HDF) cells. INS018_055 treatment resulted in a concentration-dependent reduction in the number and percentage of SA-β-gal positive cells (Supplementary Fig. 3D-F) without affecting the total cell count. Notably, siRNA-mediated TNIK knock-down phenocopied INS018_055 treatment, confirming that TNIK inhibition underlies the anti-senescence effects observed with INS018_055 (Supplementary Fig. 3G-J). These results in three distinct cellular senescence models support the potential utility of INS018_055 as a senomorphic drug by inhibiting TNIK.

### INS018_055 mitigated the replicative senescence of fibroblast

**Figure 3.**
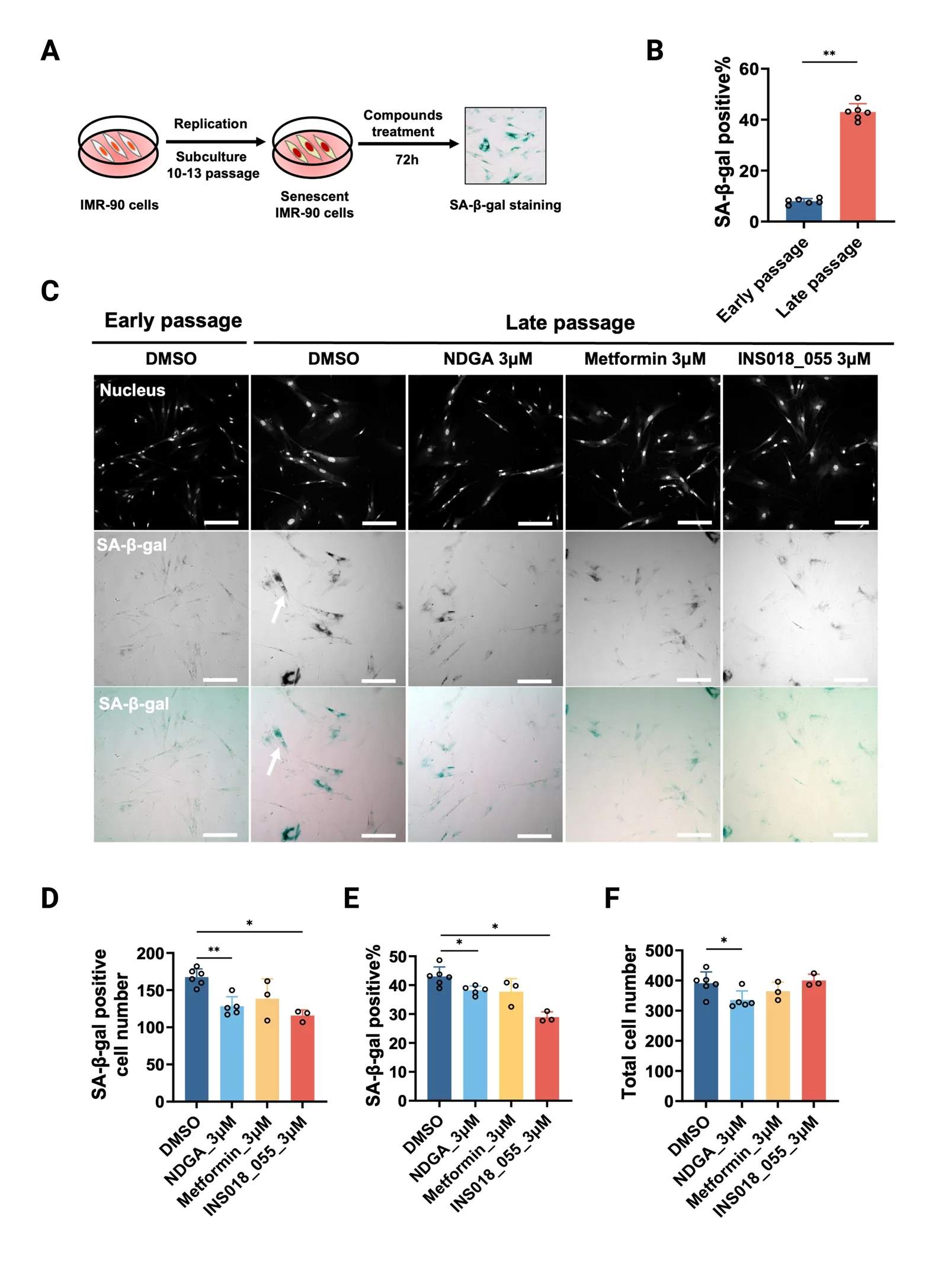
**INS018_055 inhibited fibroblast replicative senescence**. (**A**) Schematic outline of the replicative senescence model. IMR-90 cells were passaged through 10 to 13 passages to induce senescence, and cells were then treated with INS018_055 and respective controls for an additional 72 hours. Cellular senescence was measured by SA-β-gal staining. (**B**) Percentages of SA-β-gal positive cells in the early and late passage. n=6 per group; ^**^p < 0.01; unpaired two-tailed Student's t-test. (**C**) Representative staining images of replicative senescent IMR-90 cells in the early passage and late passage treated with DMSO, 3µM NDGA, 3µM metformin, and 3µM INS018_055. Top Row: Hoechst, middle row: grey-scale SA-β-gal signal, bottom row: bright field signal. White arrows are SA-β-gal positive cells. Scale bar = 200 μm. (**D**) SA-β-gal positive cell number quantitation in samples treated with DMSO, NDGA, metformin, and INS018_055 at late passage. DMSO group: n=6; NDGA group: n=5; Metformin group: n=3; INS018_055 group: n=3; ^*^p < 0.05; ^**^p < 0.01; Mann-Whitney test. (**E**) Percentage of SA-β-gal positive cells in the indicated groups. DMSO group: n=6; NDGA group: n=5; Metformin group: n=3; INS018_055 group: n=3; ^*^p < 0.05; Mann-Whitney test. (**F**) Total cell number quantitation from groups analyzed in (D). DMSO group: n=6; NDGA group: n=5; Metformin group: n=3; INS018_055 group: n=3; ^*^p < 0.05; Mann-Whitney test.

To further validate INS018_055’s senomorphic potential, we established a replicative senescence model by passaging IMR-90 cells (Fig. 3A). We found that the senescent cell percentage was significantly increased in late passage cells compared to early passage cells (Fig. 3B), supporting our experimental model for assaying effects on senescence. We then evaluated the effect of INS018_055 in parallel with two other senomorphic agents, NDGA and metformin, utilized in replicative senescence models [37, 38]. SA-β-gal analysis demonstrated that cellular senescence was significantly decreased following INS018_055, NDGA, and metformin treatment compared to DMSO-treated control cells (Fig. 3C and 3D). Consistent with findings in the chemotherapy-induced senescence models, INS018_055 treatment reduced senescent cell percentage without affecting the total cell number (Fig. 3E and 3F). Conversely, metformin treatment did not reduce SA-β-gal positive cells, while NDGA treatment induced a senolytic effect, decreasing total cell number. These collective data suggest that INS018_055 exerts a senomorphic impact on replicative senescence.

### Chronic INS018_055 treatment inhibits replicative senescence by inhibiting SASP

**Figure 4.**
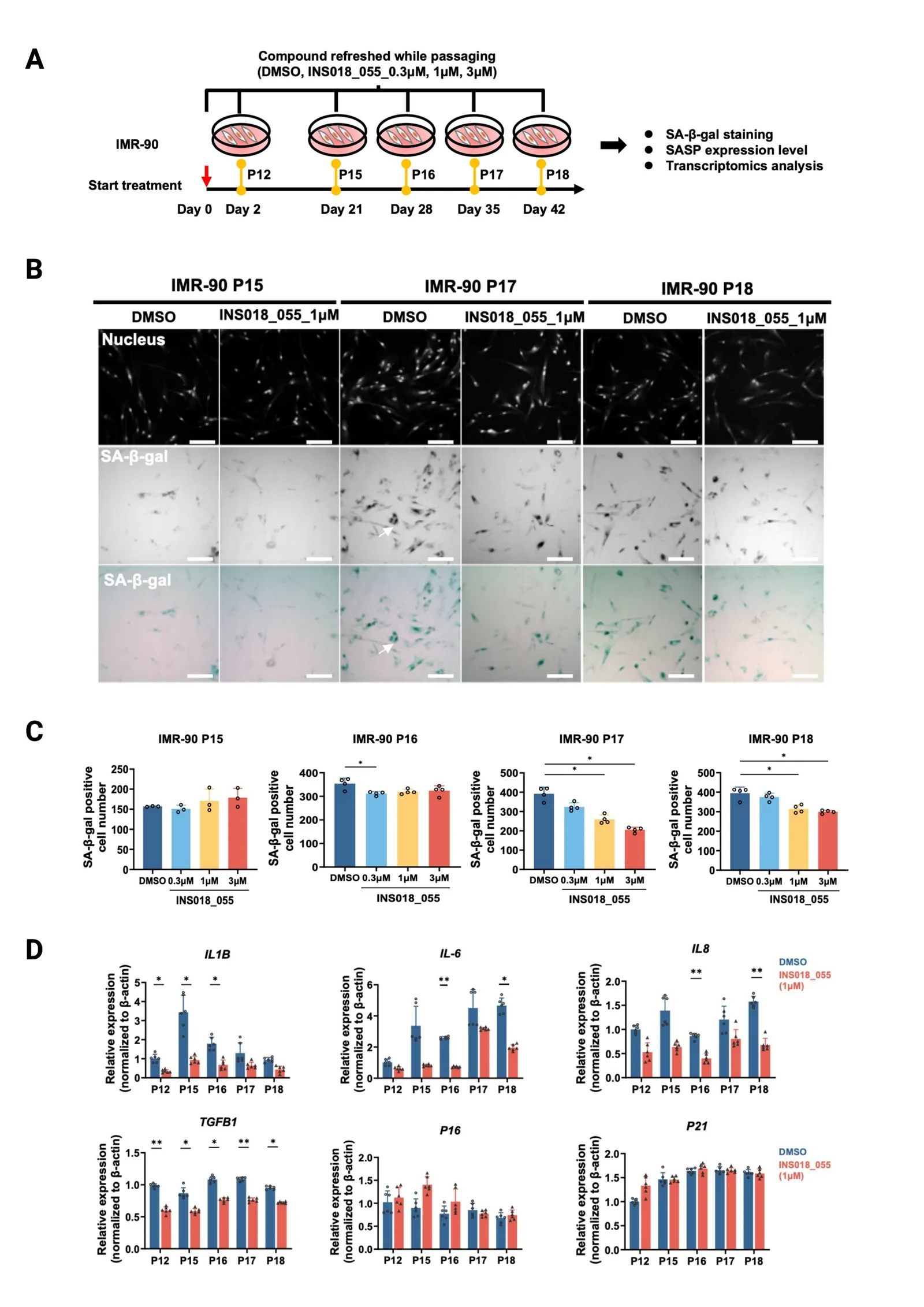
**Long-term treatment of INS018_055 prevented replicative senescence by suppressing SASP factors**. (**A**) Schematic outline of long-term replicative senescence in IMR-90 cells. IMR-90 cells were cultured for 8 weeks and passaged to induce senescence. The cells were treated with three concentrations of INS018_055 at 0.3μM, 1μM, and 3μM, as well as corresponding DMSO controls. The cellular senescence was evaluated by the SA-β-gal staining and SASP factor expression detection at different passages as indicated. Moreover, cells treated with DMSO and INS018_055 (1μM) were collected for transcriptomic analysis. (**B**) Representative staining images of senescent IMR-90 cells at P15, P17, and P18 treated with DMSO and 1µM INS018_055. Top Row: Hoechst, middle row: grey-scale SA-β-gal signal, bottom row: bright field signal. White arrows are SA-β-gal positive cells. Scale bar = 200 μm. (**C**) SA-β-gal positive cell number quantitation in samples treated with 0.3, 1, and 3μM INS018_055 at different passages. P15: n=3 per group; P16, 17, 18: n=4 per group; ^*^p < 0.05; Mann-Whitney test. (**D**) RT-qPCR analysis of cell cycle regulators and SASP. Relative expression of *P16, P21, IL1B, IL6*, *IL8 and TGFB1* were normalized against β-actin. P12, 15, 16, 17, P18: n=6 per group; ^*^p < 0.05; ^**^p < 0.01; unpaired two-tailed Student's t-test.

Given that INS018_055 treatment displayed little effect on total cell number, we hypothesized that INS018_055 mitigates cellular senescence by inhibiting SASP rather than promoting apoptosis or increasing cell proliferation. Thus, we performed long-term treatment analysis in the replicative senescence model and treated IMR-90 cells with INS018_055 at an earlier passage (two days before passage 12) (Fig. 4A). We confirmed that the senescent cell percentage increased with each passage number (Supplementary Fig. 4A) through terminal passage 18. However, INS018_055 treatment failed to extend cell proliferation capacity as measured by population doubling over time (Supplementary Fig. 4B). As expected, INS018_055 treatment reduced the number of senescent cells from P16 to the experiment's conclusion at P18 (Fig. 4B and 4C). We performed qPCR analysis to detect the expression of SASP-associated cytokines and cell cycle arrest genes. Intriguingly, INS018_055 treatment mitigated increases in pro-inflammatory SASP factors *IL1B*, *IL6*, and *IL8* relative to DMSO-treated controls (Fig. 4D). Consistent with our previous findings [32], INS018_055 dramatically inhibited the expression of *TGFB1* (Fig. 4D). However, cell cycle-associated genes *P16* and *P21* were unchanged. Thus, our findings indicate that long-term treatment with INS018_055 prevents replicative senescence, likely via suppression of SASP, without inducing cell death in lung fibroblasts.

### INS018_055 attenuated cellular senescence through regulation of aging-related pathways

**Figure 5.**
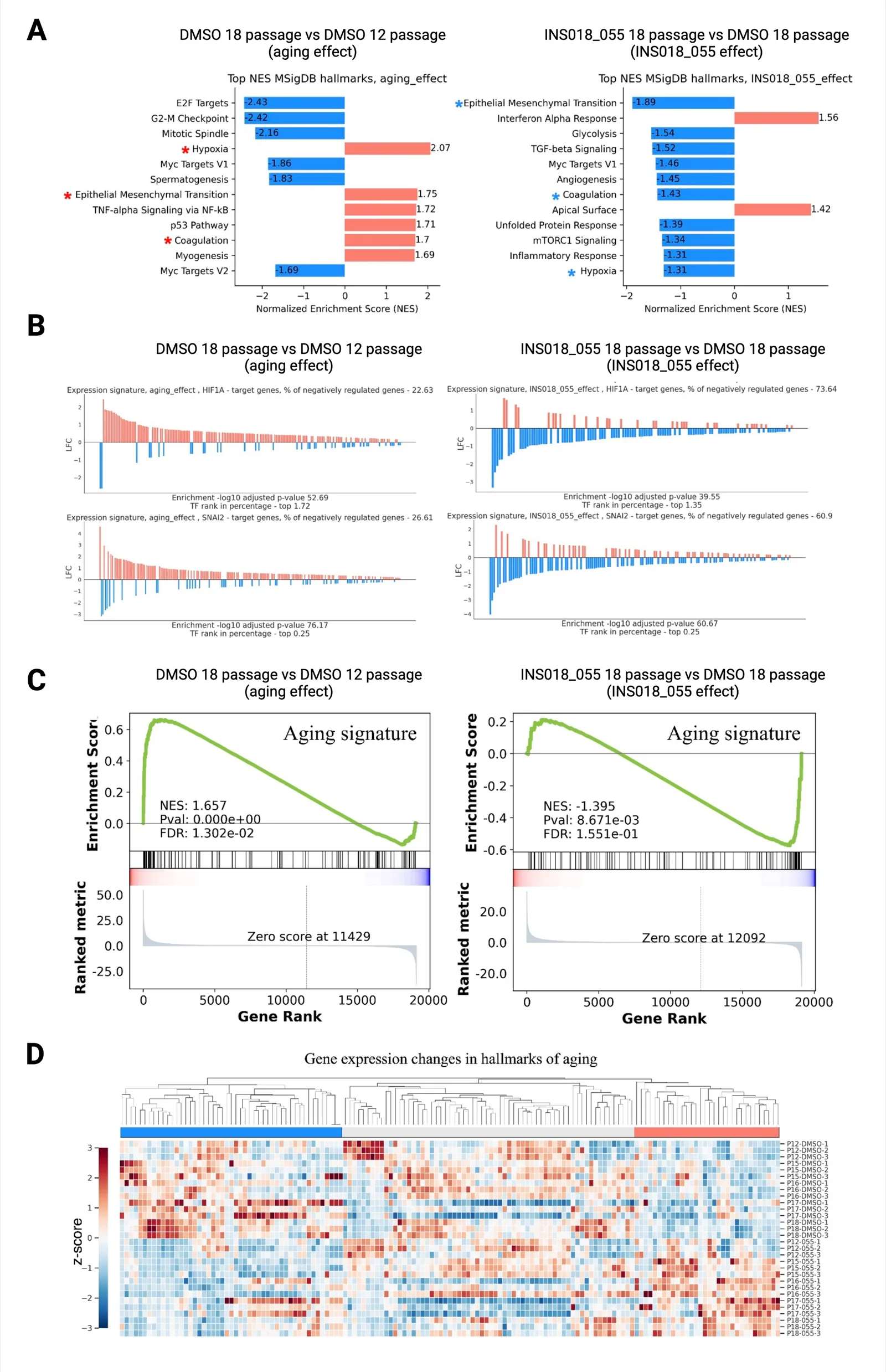
**Transcriptomic analysis of INS018_055-treated senescent cells**. (**A**) GSEA of the MSigDB hallmarks in aging (DMSO 18 passage vs. DMSO 12 passage) and compound-treated groups (INS018_055 18 passages vs. DMSO 18 passage). (**B**) Transcription factor enrichment analysis of HIF1A and SNAI2 regulated genes in aging and compound treated groups. (**C**) GSEA of the aging signature in aging (DMSO 18 passage vs. DMSO 12 passage) and compound treated (INS018_055 18 passages vs. DMSO 18 passage) groups. (**D**) Gene expression heatmap annotated to the hallmark of “Aging” in DMSO or INS018_055 treatment groups.

To gain insights into the mechanisms underlying INS018_055's effects on the aging process, we conducted bulk RNA-seq analysis on IMR-90 cells treated with INS018_055 or DMSO in the replicative senescence model. We observed that five hallmarks of cell proliferation-related gene sets (“E2F Targets”, “G2-M Checkpoint”, “Mitotic Spindle”, “MYC Targets V1 and V2”) from MsigDB [39] were negatively enriched in P18 DMSO-treated group compared to that of P12 group (Fig. 5A). This indicated that P18 cells reach a stable proliferative arrest state. Conversely, hallmarks associated with senescence-related gene sets such as “Hypoxia” [40, 41], “Epithelial mesenchymal transition (EMT)” [42], “TNF-alpha signaling via NF-KB”, “P53 pathway” [43], and “Coagulation” [44] were positively enriched in P18 cells (Fig. 5A, left). Furthermore, aged DMSO-treated cells were enriched for three hallmarks of aging gene sets (“Hypoxia,” “Epithelial-mesenchymal transition,” and “coagulation”) compared to INS018_055-treated aged cells (Fig. 5A, right). Treatment with INS018_055 also downregulated genes involved in “TGF-β signaling” and “Inflammatory response” pathways, gene sets positively associated with cellular senescence. This enrichment analysis demonstrates that the INS018_055 administration inhibits several transcriptional programs that promote cellular senescence. Transcription factor enrichment analysis identified HIF1A and SNAI2 as the key transcription factors enriched with differentially expressed genes, known to be involved in hypoxia, extracellular matrix, and EMT processes. Most of the significantly perturbed genes regulated by HIF1A and SNAI2 were upregulated in aged cells and downregulated after INS018_055 treatment (Fig. 5B).

Next, we calculated the enrichment of the validated SenMayo gene set of 125 aging-related genes [45], which includes SASP factors (n=83), transmembrane (n=20), and intracellular (n=22) proteins, across our samples. We analyzed DMSO passage 18 vs. DMSO passage 12 for SenMayo GSEA (Fig. 5C, left), which was enriched in the DMSO passage 18 group (NES score of 1.657, p < 0.05, FDR=1.302E02). In comparing INS018_055 passage 18 vs. DMSO passage 18, we observed an enrichment of the senescence gene set in the DMSO passage 18 cell population (Fig. 5C, right, NES=-1.395, p < 0.05, FDR=1.551E01), indicating INS018_055 treatment mitigated SenMayo transcriptional changes. Thus, GSEA was consistent with the reduction of SA-β-gal-positive senescent cell numbers following INS018_055 treatment, supporting the conclusion that INS018_055 is a senescence-mitigating agent.

A heatmap of aging hallmark genes from passages 12 to 18 in DMSO- and INS018_055-treated cells (Fig. 5D) shows three archetypes of gene expression patterns: genes with passage-correlated expression downregulated early (P12) by INS018_055, genes whose expression correlated with passage but not treatment, and genes upregulated by INS018_055 (Fig. 5D).

We matched changes in aging-related genes to the hallmarks of aging using a classification proposed by Pun and colleagues [5]. DMSO-treated cells exhibited increased expression of aging-related gene sets related to most of the 12 hallmarks of aging at passage 18 compared to passage 12 (Supplementary Fig. 5), with the exceptions of epigenetic shift, genomic instability, and telomere attrition. INS018_055 treatment induced a stable and sustained downregulation of genes related to nearly all hallmarks of aging, including extracellular matrix stiffness, cellular senescence, and altered intercellular communications.

### INS018_055 ameliorated SASP via regulation of TNIK-associated crosstalk of WNT and TGF-β signaling

**Figure 6.**
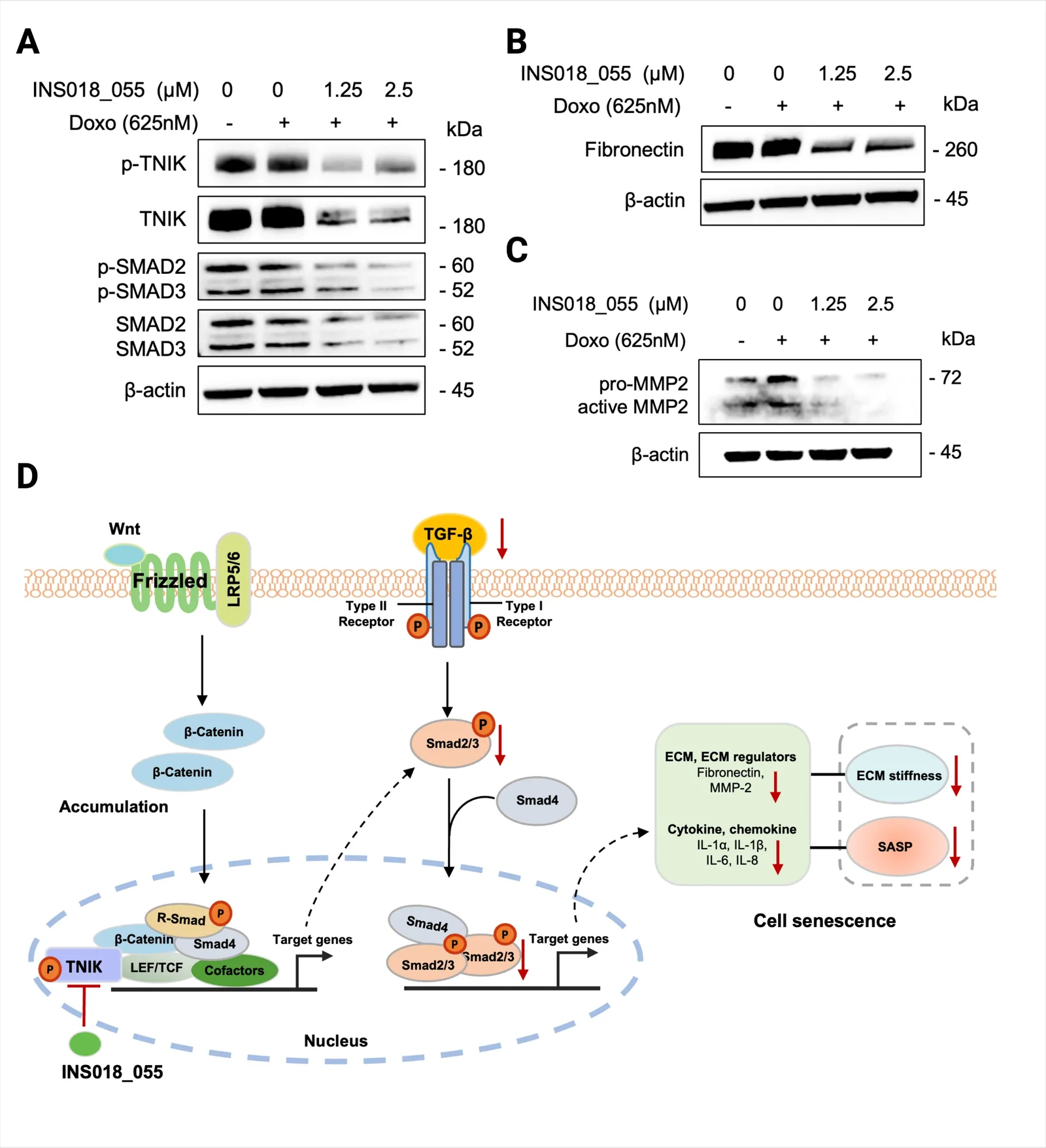
**INS018_055 reduced cellular senescence by mitigating ECM deposition and SASP-associated inflammatory signaling**. (**A**) TNIK, SMAD2, SMAD3, and their respective phosphorylated protein in cells treated with 72h doxorubicin measured by western blot at the indicated condition. β-actin served as a loading control. (**B-C**) Western blot analysis of fibronectin and matrix metallopeptidase 2 (MMP2), normalized to β-actin. (**D**) Proposed mechanism for the senomorphic effect of INS018_055 in senescent cells.

As our RNA sequencing data revealed that INS018_055 modulates extracellular matrix and intercellular communication-related gene sets, we hypothesized that INS018_055 reduces SASP by inhibiting TGF-β signaling. In line with this hypothesis, INS018_055 treatment reduced the abundance of TNIK and its active form, p-TNIK (Ser764), in the doxorubicin-induced IMR-90 senescence model (Fig. 6A). Reduced p-TNIK likely impinges on its nuclear translocation, suppressing the transcriptional activity of the TNIK/TCF4/β-catenin complexes. Indeed, we also observed the downregulation of p-SMAD2/3 and total SMAD2/3 following INS018_055 treatment (Fig. 6A). These results suggest that INS018_055 may exert its inhibitory effect via SMAD-dependent signaling, reducing TGF-β gene transcription in an autocrine manner.

Next, we evaluated INS018_055’s impact on ECM components regulated by the TGF-β/p-SMAD pathway. INS018_055 treatment significantly reduced fibronectin and matrix metallopeptidase 2 (MMP2) abundance, suggesting a protective role in ameliorating ECM deposition (Fig. 6B-C). We concluded that INS018_055 exerts its senomorphic effects partly by inhibiting the transcriptional activity of TNIK/TCF/LEF and β-catenin nuclear complexes. This attenuation subsequently results in diminished expression of critical ECM constituents and SASP-associated cytokines through the dysregulation of the TGF-β/p-SMAD signaling pathway (Fig. 6D).

## DISCUSSION

Drugs that slow down or even reverse the aging process are likely to modulate the progression of age-related diseases. However, the discovery of longevity therapeutics often requires novel drugs to first be developed to target specific diseases in order for the drug discovery program to be commercially viable. The discovery and prioritization of TNIK as a target, for example, involved AI-based integration and discovery of the biomarkers and hallmarks of aging and fibrosis [32]. Having previously implicated TNIK in the cellular senescence hallmark of aging utilizing a computational approach [10], we report here validation of the senomorphic potential of a recently developed anti-fibrotic TNIK inhibitor, INS018_055, in multiple fibroblast senescence models: chemotherapy-induced senescence and replicative senescence. Knockdown of TNIK phenocopied the anti-senescence effects of INS018_055 treatment in a chemotherapy-induced senescence model, confirming the TNIK-mediated phenotypic effects of the treatment.

An automated robotics lab conducted these *in vitro* studies and subsequent post-treatment transcriptomic analyses (Fig. 1 and 2A). An automatic high-content imaging-based analysis pipeline for senotherapeutics screening and validation was developed for this study. In comparison with traditional analysis methodology, the developed pipeline is capable of identifying two different types of senotherapeutics, senomorphics and senolytics, by analyzing thousands of cells based only on the readout of SA-β-gal positivity free of human biases. Moreover, the analysis could be performed in a high-throughput way which can significantly accelerate the process of senotherapeutic identification. It is noteworthy that this automatic high-content imaging-based analysis pipeline was validated in different senescence models with several well-known senomorphics and senolytics, suggesting the methodology is sensitive, robust and reproducible.

INS018_055 treatment reduced SASP-associated proinflammatory release of *IL6, IL8, IL1A*, and *IL1B* (Fig. 2F, 4D) and reduced senescent cell percentages relative to DMSO-treated cells (Fig. 2B-E, 3C-F and 4B-C). Notably, treatment had a minimal effect on total cell numbers, indicating a negligible effect on cell viability. RNA sequencing analysis of DMSO-treated and INS018_055-treated cells revealed the senomorphic activity of INS018_055 was accomplished by reversing multiple gene sets associated with aging, such as TGF-β signaling, hypoxia, EMT, and inflammation, among others (Fig. 5). HIF1A was the top TF whose regulon was downregulated with INS018_055 treatment. HIF1A and its upstream regulatory degradation factors PHD1-3 have been implicated in aging and senescence [46]. We previously developed a selective, tissue-restricted inhibitor against PHD1/2 that restores mucosal barrier function in models of IBD characterized by a dysfunctional hypoxic gradient [47].

Further investigation revealed that TNIK inhibition in senescent cells attenuated TGF-β signaling and downstream production of critical ECM components like fibronectin and MMP-2 (Fig. 6A-C). Thus, INS018_055 achieves its senomorphic effects in two parallel processes: i. inhibition of SASP-associated proinflammatory cytokine production and ii. inhibition of TGF-β signaling to reduce downstream pro-fibrotic programs that have been implicated in promoting cellular senescence (Fig. 6D). TGF-β signaling plays a crucial role in cellular senescence and the aging process. Excessive TGF-β signaling in multiple cell types has been shown to accelerate cellular senescence, driving pathologies involving fibrosis. Specifically, TGF-β signaling promotes the transcription of senescence-related cellular programs, including SASP, reactive oxygen species (ROS), DNA damage repair, telomere regulation, unfolded protein response (UPR), and autophagy [48]. TGF-β1 has been shown to promote SASP by driving ﬁbroblast activation, collagen deposition, and ECM remodeling. These observations are directly relevant to our observations wherein we report INS018_055 treatment impinges upon critical events during SASP development, including TGF-β1 production, pro-inflammatory factor secretion, and ECM deposition. Since SASP can disrupt tissue homeostasis and promote chronic inflammation, INS018_055’s inhibition of SASP-related proinflammatory cytokine production may help alleviate chronic low-grade inflammation observed in degenerative diseases.

This study also explains how INS018_055-mediated inhibition of TNIK and SMAD proteins modulates their downstream transcriptional activities (Fig. 6). SMADs have been reported to interact with the nuclear complexes of TNIK/TCF/LEF and β-catenin/p-SMAD, thus regulating their transcriptional activity [49]. β-catenin signaling is required for TGF-β-induced expression of ECM proteins by fibroblasts [50]; in turn, TGF-β/SMAD signaling is indispensable to the induction of genes related to ECM remodeling by speciﬁc WNT ligands. INS018_055’s inhibitory effect on these nuclear transcription complexes likely mediates the crosstalk between the Wnt/β-catenin and TGF-β/p-SMAD signaling pathways. This observation is notable as Wnt/β-catenin pathway activation promotes TGF-β/SMAD2 signaling during myofibroblast differentiation in fibrotic pathologies [51]. Wnt/β-catenin signaling also promotes collagen (multiple isoforms) and fibronectin synthesis and deposition to promote fibrotic progression [49]. Based on these observations, we conclude that INS018_055-mediated inhibition of TNIK likely impinges upon Wnt/β-catenin pathway signaling. This negative feedback cycle downregulates TGF-β signaling in an autocrine loop, significantly reducing ECM remodeling and inflammation during cellular senescence (Fig. 6D).

Generative AI has been indispensable to aging-related drug discovery efforts due to its efficiency and accuracy in identifying unappreciated drug targets while simultaneously aiding in the design of therapeutic interventions [52]. As INS018_055 became the first AI-designed drug to enter clinical testing for an AI-discovered disease-associated target [32] following success in multiple preclinical murine models of fibrosis and phase 1 clinical testing, it is likely that future studies will implement this approach for many other disease indications. While AI analysis platforms will surely benefit the identification of unappreciated aging-related biomarkers, these discoveries will also depend upon AI-driven *in vitro* and *in vivo* validation of these findings in a methodology similar to the one used by Ren and colleagues [32]. Indeed, the AI-powered robotics pipeline for *in vitro* analysis used in this study is one such tool that, together with other proof-of-concept AI-powered automated chemical and drug synthesis and testing platforms [53-55], have demonstrated utility in diverse disease settings to streamline the time and costs associated with preclinical testing during drug development.

Importantly, in addition to cellular senescence, one pivotal hallmark studied here, the AI-driven robotics platform identified other aging hallmarks such as epigenetic and mitochondrial dysfunction [3]. Epigenetic regulation plays an important role in the aging and pathogenesis of age-related diseases [56]. Recently, we have developed a multimodal transformer by combining transcriptomic and DNA methylation data from different age groups in different species to accelerate the target identification of age-related diseases [33]. Mitochondrial dysfunction in the aging process not only leads to cellular energy crises but also inflammation, and AI-driven phenotypic characterization of mitochondria could accelerate mitochondrial protector identification [57]. Target prioritization of candidates involved in biological aging-related processes as well as those pertinent to aging-related disorders like organ fibrosis will aid in identifying dual-purpose targets that are less likely to fail when translating preclinical findings [14, 27].

Senescent cells accumulate with age in mammals and display a pro-inflammatory SASP, which fuels multiple age-related disorders, including IPF [58]. IPF predominantly affects aged adults, with a higher incidence and prevalence in individuals over 60 years of age [59, 60]. This chronic progressive lung disease poses a poor prognosis, often leading to death within 2 to 5 years if left untreated [61]. While antifibrotic drugs such as nintedanib and pirfenidone can mitigate the decline in lung function, their continued efficacy in IPF patients over 75 years old remains unclear [62]. Targeting senescent cells may offer an alternative therapeutic strategy to counter this progressive aging-related pathology.

INS018_055 offers distinct advantages as a senomorphic drug over senolytic drugs. Senescent cells exhibit heterogeneity in their transcriptional landscapes, metabolic activities, and SASP profiles, posing a significant challenge in targeted interventions without affecting healthy cells [7]. Although some senolytics, such as dasatinib plus quercetin (D+Q), could alleviate symptoms, improve the physical function in rodent IPF models, and be generally well-tolerated in clinical trials [9, 12], other senolytic drugs like ABT-263 (navitoclax) eliminate senescent cells but exhibit cytotoxicity in non-senescent cells, leading to undesirable side effects such as thrombocytopenia and neutropenia [63]. Completely eradicating all senescent cells may disrupt crucial biological processes, including tissue repair and tumor prevention [64]. Given the complexities surrounding senescent cells, senomorphics present a superior treatment strategy by attenuating the pathological and pro-inflammatory SASP without inducing cell senolysis [7]. Classical senomorphics, such as rapamycin and metformin, exert their effects by selectively targeting signaling pathways including NF-kB, mTOR, IL-1a and p38 MAPK, inhibiting SASP [65, 66].

Recently, several promising senomorphics have been identified through compound screening in senescent cell models, including regorafenib, a multityrosine kinase inhibitor [67]. Regorafenib, approved for treating metastatic colorectal cancer refractory to standard chemotherapy, displays multi-kinase inhibitory activity. Although Regorafenib can inhibit the proliferation of cancer cells in vitro, *Jung-Jin* et al. demonstrated that fibroblast viability was only inhibited at concentrations above 2 µM [67]. In our previous study, INS018_055 was shown to target several fibrosis-related kinases including TNIK, ALK4, TGFBR1, and DDR1 [32]. In fibroblasts, INS018_055 only suppresses cell viability at a much higher concentration of 84.3-µM. Moreover, rapamycin, which was approved by FDA in 1999 to prevent transplant rejection, inhibited cell proliferation in non-senescent fibroblasts at the low concentration of 50 nM (Supplementary Fig. 2E). However, in senescent fibroblasts, neither rapamycin nor INS018_055 suppresses cell proliferation. These data suggest that INS018_055 could be better tolerated than other senomorphics such as regorafenib and rapamycin.

Regorafenib, rapamycin, and INS018_055 significantly attenuated the phenotypes of senescent fibroblasts, including SA-β-gal positivity and SASP, especially IL-6 and IL-8 expression. Additionally, we found that INS018_055 displayed similar inhibitory activity on SA-β-gal positivity of senescent fibroblasts as rapamycin (Fig. 2C-E, p < 0.05, Mann-Whitney test) and even higher inhibitory activity than NDGA and metformin (Fig. 3E, p < 0.05, Mann-Whitney test). *In vivo* studies have demonstrated that treatment with these compounds could attenuate cellular senescence in tissues, improving organ function in disease models. However, the clinical efficacy of these compounds in age-related diseases still requires investigation in future clinical trials.

Although rapamycin exhibits broad inhibition of cellular senesce in different cell types *in vitro* [37], there is limited evidence to show its efficacy in age-related disease treatment and aging. Recently, rapamycin was summarized as having no significant effects on the endocrine, muscular, or neurological systems in human [68]. Notably, muscle loss or wasting, also known as sarcopenia, is a detrimental factor for elderly people, and this condition occurs in approximately 50% of individuals over 80 [69]. In contrast to the hyperactivation of TGF-β signaling to contribute to muscle atrophy, PI3K/AKT/mTOR signaling is an important pathway for protein synthesis and muscle growth [69]. Rapamycin administration in humans is reported to block the contraction-induced increase in skeletal muscle protein synthesis by targeting the mTOR signaling. Together, these findings suggest that the effects of rapamycin or its derivatives on aging or age-related diseases need further clinical studies.

We previously demonstrated INS018_055’s favorable safety and tolerability profiles in two independent Phase I clinical trials and a Phase IIa clinical trial (NCT05938920). Importantly, the secondary efficacy endpoint showed dose-dependent improvements in forced vital capacity (FVC), with the largest improvement observed in the 60mg QD cohort. A parallel Phase IIa study (NCT05975983) is currently underway in the United States to further validate these findings. The accumulation of senescent cells in the lungs of IPF patients is a major driver of pathogenesis. In the current study, INS018_055 exhibited comparable senomorphic activity with well-known senomorphics such as rapamycin *in vitro* by significantly inhibiting SA-β-gal activity and SASP that should be further validated in future *in vivo* studies. Furthermore, INS018_055 has potential dual-purpose utility in acting upon anti-aging and anti-fibrosis pathways often intertwined during senescent cell accumulation [32].

In addition to lung, TNIK is extensively expressed in the heart, brain, and skeletal muscle [70], suggesting dysfunction of TNIK may contribute to age-related diseases in these organs, such as cardiovascular diseases, neurodegenerative disease, and muscle atrophy. Pulmonary artery hypertension (PAH) is a rare, progressive, and fatal cardiopulmonary condition characterized by increased pulmonary arterial pressure, structural changes in the pulmonary circulation, and the formation of vaso-occlusive lesions [71]. The pathogenesis of PAH involves the imbalance between TGF-β signaling and BMP signaling [72].The hyperactivation of TGF-β signaling leads to the hyperproliferation of vascular smooth muscle cells, dysfunction of endothelial cells including apoptosis, proliferation, and senescence, and abnormal ECM deposition, etc. [73]. The majority of available treatments for PAH focus on vasodilation by targeting nitric oxide signaling [74], endothelin signaling [75], and prostacyclin signaling [76]. Notably, Sotatercept, a fusion protein that acts as a ligand trap for TGF-β signaling members approved by the FDA in March 2024, induced a greater improvement in exercise capacity of PAH patients by suppressing the GDF8/ACTIVIN A-activated TGF-β signaling [77]. Based on the promising results of suppressing TGF-β signaling and clinical safety, INS018_055 could be further explored in related disease areas such as PAH.

Our findings collectively demonstrate the potential of INS018_055 as a senomorphic drug in attenuating cellular senescence through the suppression of various aging processes. This study further strengthens the potential of INS018_055 as a longevity therapeutic by implicating TNIK in the cellular senescence hallmark of aging. Using AI-powered drug discovery [32] and a fully automated robotics laboratory, we achieved automated, efficient sample handling and data generation, facilitating target discovery, compound design, clinical trial prediction, mechanistic aging research, and biomarker identification. The role of INS018_055 in the attenuation of senescence may provide an explanation for its beneficial effects in preclinical and clinical investigations in IPF. However, *in vivo* data and validation of biomarkers supporting its use for anti-aging therapy in clinical settings are still needed. Taken together, this research highlights the novel role of TNIK in cellular senescence and new senomorphic applications for INS018_055, in addition to its anti-fibrotic properties that may inform future efforts to treat age-related diseases.

## Supplementary Materials

The Supplementary data can be found online at: www.aginganddisease.org/EN/10.14336/AD.2024.1492.
