# Supplementary material for: AI-Driven Robotics Laboratory Identifies Pharmacological TNIK Inhibition as a Potent Senomorphic Agent
Source: Aging Dis. 2025 Feb 13;17(1):432–51. doi: 10.14336/AD.2024.1492 (PMC12727053; doi:10.14336/AD.2024.1492)
Supplement: Supplementary file 1 — The Supplementary data can be found online at: www.aginganddisease.org/EN/10.14336/AD.2024.1492. [file AD-17-1-432-s.pdf]

SUPPLEMENTARY DATA

AI-Driven Robotics Laboratory Identifies Pharmacological TNIK Inhibition as a Potent Senomorphic Agent

Qiuqiong Tang, Deyong Xiao, Alexander Veviorskiy, Ying Xin, Sarah W.Y. Lok, Fadi E. Pulous, Peiran Zhang, Yunfeng Zhu, Yongming Ma, Xiao Hu, Shoulai Gu, Chenting Zong, Sabina Mukba, Mikhail Korzinkin, Frank W. Pun, Man Zhang, Alex Aliper, Lijuan Wu, Feng Ren, Li Zhang, Alex Zhavoronkov

© 2024. Tang Q et al. Published online at http://www.aginganddisease.org/EN/10.14336/AD.2024.1492

SUPPLEMENTARY DATA

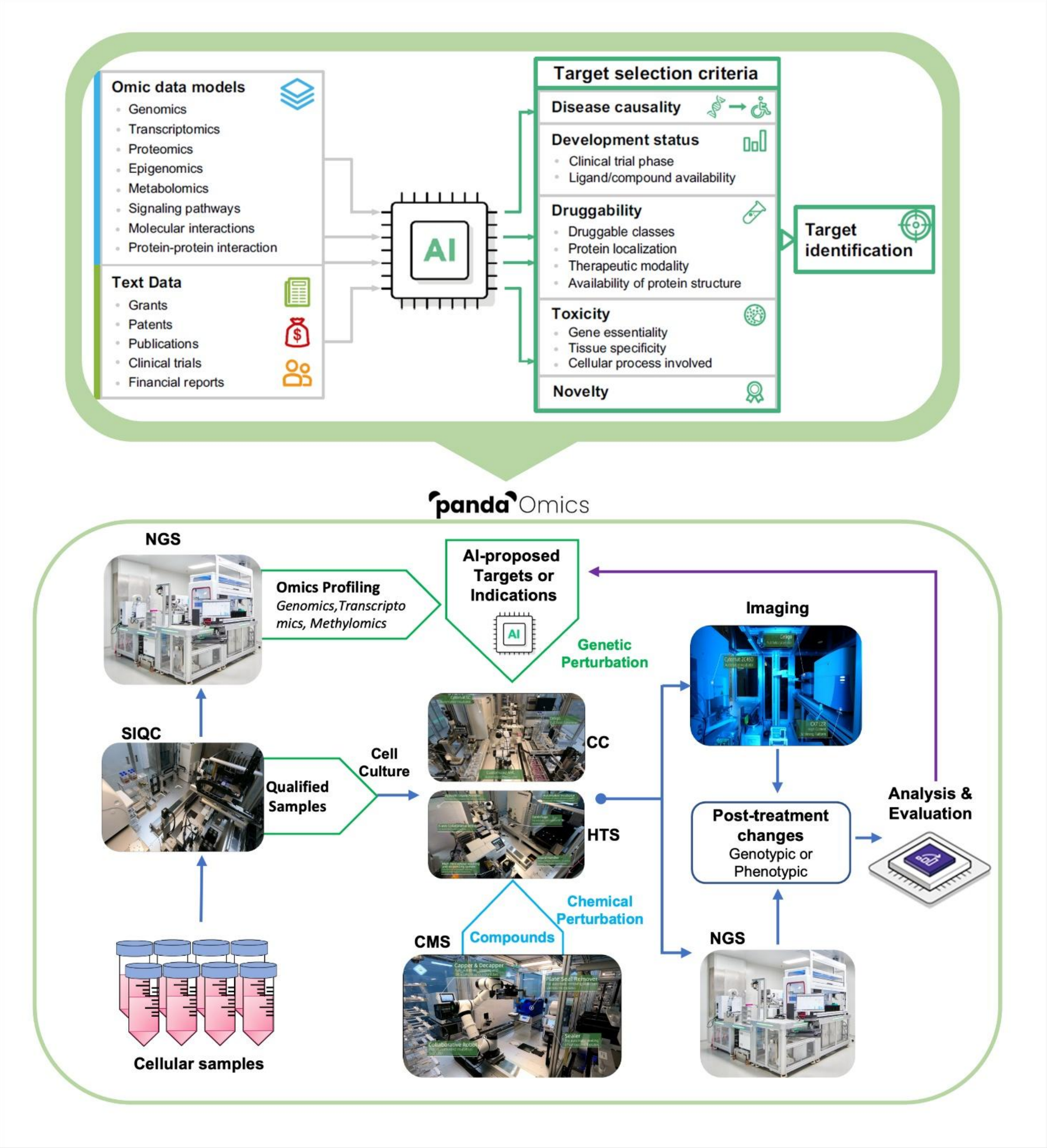

Supplementary Figure 1. A typical robotic lab workflow.

© 2024. Tang Q et al. Published online at http://www.aginganddisease.org/EN/10.14336/AD.2024.1492

SUPPLEMENTARY DATA

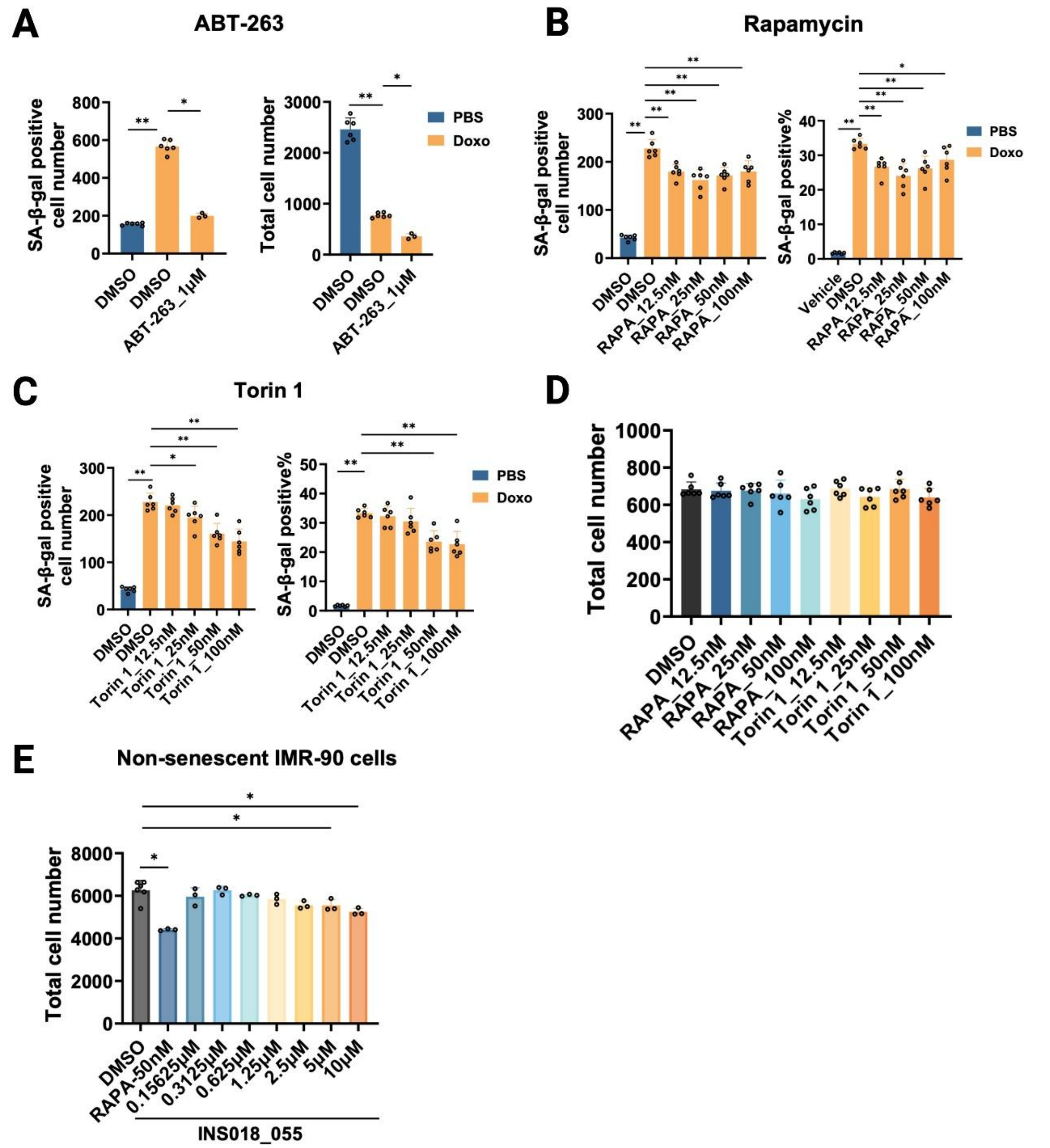

Supplementary Figure 2. ABT-263 and mTOR inhibitors exhibited senolytic and senomorphic activities in chemotherapy-induced senescence models. (**A**) Quantitation of SA-β-gal-positive senescent cells and total cell numbers in ABT-263-treated cells. DMSO group: n=6; Doxo group: n=6; ABT-263 group: n=3; $^{*}p < 0.05$; $^{**}p < 0.01$; unpaired two-tailed Student's t-test for the comparison between DMSO and Doxo group and Mann-Whitney test for the comparison between Doxo and ABT-263 group. (**B**) Quantitation of SA-β-gal-positive senescent cells and the percentage of SA-β-gal-positive cells in Rapamycin-treated cells. n=6 per group; $^{*}p < 0.05$; $^{**}p < 0.01$; unpaired two-tailed Student's t-test. (**C**) Quantitation of SA-β-gal-positive senescent cells and the percentage of SA-β-gal-positive cells in Torin 1-treated cells n=6 per group; $^{*}p < 0.05$; $^{**}p < 0.01$; unpaired two-tailed Student's t-test. (**D**) Total cell numbers for groups presented in (**B-C**). n=6 per group; unpaired two-tailed Student's t-test. (**E**) Evaluation of non-senescent IMR-90 cells in Rapamycin or INS018_055-treated groups. DMSO group: n=6; Rapamycin group: n=3; INS018_055 group: n=3; $^{*}p < 0.05$; Mann-Whitney test.

© 2024. Tang Q et al. Published online at http://www.aginganddisease.org/EN/10.14336/AD.2024.1492

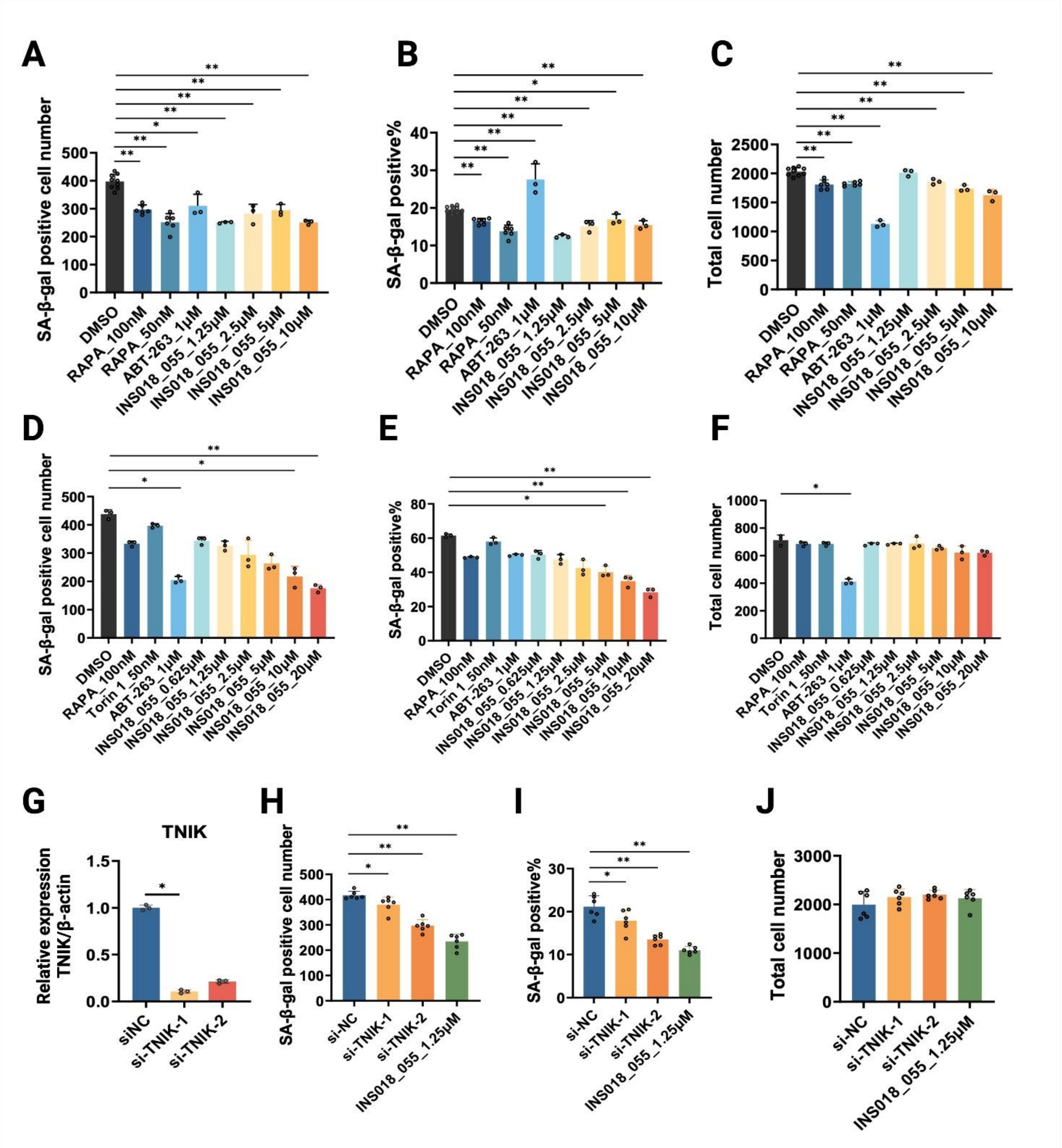

Supplementary Figure 3. Pharmacological TNIK inhibition or siRNA-mediated TNIK knockdown induced comparable senomorphic effects in chemotherapy-induced senescence models. (**A-C**) TNIK inhibition with INS018_055 induced a senomorphic effect on doxorubicin-induced senescent MRC-5 cells. Quantitation of SA-β-gal positive cell number, percentage of SA-β-gal positive cells, and total cell number in samples treated with DMSO, RAPA, and INS018_055 at indicated concentrations. DMSO group: n=9; ABT-263 group: n=3; Rapamycin groups: n=6; INS018_055 groups: n=3; $^{*}p < 0.05$; $^{**}p < 0.01$; unpaired two-tailed Student's t-test for the comparisons between DMSO and Rapamycin groups and Mann-Whitney test for other comparisons (n<6). (**D-F**) TNIK inhibition with INS018_055 induced a senomorphic effect in primary human dermal fibroblast (HDF) cells induced by doxorubicin. Quantitation of SA-β-gal positive cell number, percentage of SA-β-gal positive cells, and total cell number in samples treated with DMSO, RAPA, and INS018_055 at indicated concentrations. Rapamycin and torin-1 served as senomorphic controls, and ABT-263 was a senolytic control. n=3 per group; $^{*}p < 0.05$; $^{**}p < 0.01$; Kruskal-Wallis test. (**G-J**) TNIK knock-down promoted senomorphic effects on doxorubicin-induced senescent IMR-90 cells. (**G**) IMR-90 cells were transfected with non-targeting siRNA (siNC) or siRNA targeting TNIK for 72 hours. Cells were then collected, and quantitative RT-PCR was performed to evaluate the TNIK knock-down efficiency.

© 2024. Tang Q et al. Published online at http://www.aginganddisease.org/EN/10.14336/AD.2024.1492

n=3 per group; *p < 0.05; Kruskal-Wallis test. (**H-J**) IMR-90 cells were treated with siNC or siTNIK for 16 hours. Subsequently, the cells were exposed to doxorubicin for 2 hours. Cell medium was then replaced with fresh complete culture medium or medium supplemented with INS018_055 at a final concentration of 1.25μM for 72 hours. Quantitation of SA-β-gal positive cells, the percentage of SA-β-gal positive cells, and the total cell count in samples treated with siNC, siTNIK, or INS018_055 at 1.25 μM. n=6 per group; *p < 0.05; **p < 0.01; unpaired two-tailed Student's t-test.

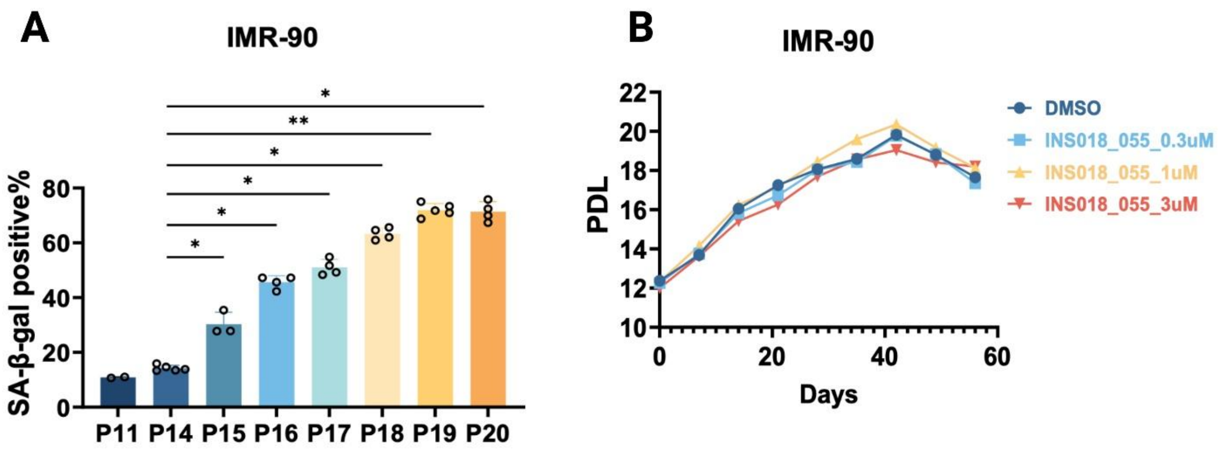

Supplementary Figure 4. Evaluation of long-term treatment of INS018_055 in the replicative senescence model. (**A**) Quantitation of the percentage of senescent cells at each cell passage number. P11: n=2, P14: n=5, P15: n=3; P16, 17, 18, 20: n=4, P19: n=5; *p < 0.05; **p < 0.01; Mann-Whitney test. (**B**) Cell population doubling level (PDL) was analyzed at the indicated passage number.

SUPPLEMENTARY DATA

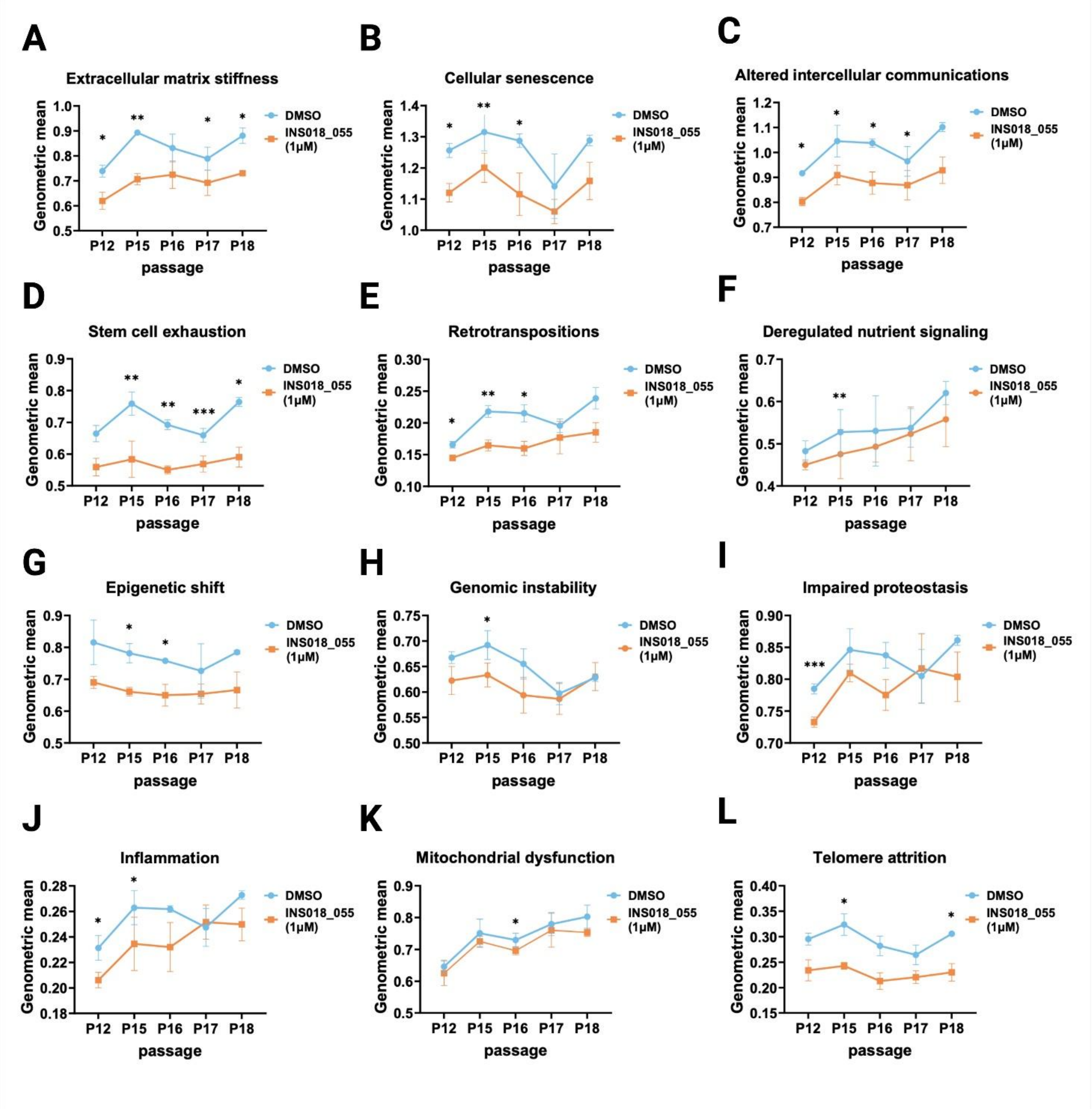

Supplementary Figure 5. Effects of INS018_055 on 12 hallmarks of aging-related genes. Gene expression changes in hallmarks of aging between the passages for treated (INS018_055) and untreated (DMSO) aged cells. For each sample, the geometric mean of gene expression comprising the investigated pathway was computed. A paired t-test was conducted using the stats.ttest_rel function from the scipy package. (n=3 per group. $^{*}p < 0.05$, $^{**}p < 0.01$, $^{***}p < 0.001$).

Supplementary Video 1. Introduction of the six-generation robotics lab.

SUPPLEMENTARY DATA

Supplementary Table 1. Cohen’s d analysis for comparisons.

Comparison	Cohen's d value
**Figure 2**	
**(C) SA-β-gal positive cell number**	
DMSO vs RAPA_100nM	2.449
DMSO vs INS018_055_1.25μM	1.899
DMSO vs INS018_055_2.5μM	2.146
DMSO vs INS018_055_5μM	2.449
DMSO vs INS018_055_10μM	2.449
**(D) SA-β-gal positive%**	
DMSO vs RAPA_100nM	2.449
DMSO vs INS018_055_0.3125μM	1.899
DMSO vs INS018_055_0.625μM	1.899
DMSO vs INS018_055_1.25μM	2.449
DMSO vs INS018_055_2.5μM	2.449
DMSO vs INS018_055_5μM	2.449
DMSO vs INS018_055_10μM	2.449
**(E)Total cell number**	
DMSO vs INS018_055_0.625μM	1.899
DMSO vs INS018_055_2.5μM	2.449
**(F)**	
**IL6:** DMSO vs INS018_055	6.925
**IL8:** DMSO vs INS018_055	3.773
**TGFB1:** DMSO vs INS018_055	2.470
**IL1A:** DMSO vs INS018_055	3.893
**IL1B:** DMSO vs INS018_055	3.146
**Figure 3**	
**(B) SA-β-gal positive%**	
Early passage vs Late passage	14.396
**(D) SA-β-gal positive cell number**	
DMSO vs NDGA_3μM	2.928
DMSO vs INS018_055_3μM	2.449
**(E)Total cell number**	
DMSO vs NDGA_3μM	1.760
**(F) SA-β-gal positive%**	
DMSO vs NDGA_3μM	2.049
DMSO vs INS018_055_3μM	2.449
**Figure 4**	
**(C) SA-β-gal positive cell number**	
**P16:** DMSO vs INS018_055_0.3μM	2.828
**P17:** DMSO vs INS018_055_1μM	2.828
**P17:** DMSO vs INS018_055_3μM	2.828
**P18:** DMSO vs INS018_055_1μM	2.828
**P18:** DMSO vs INS018_055_3μM	2.828
**(D) IL1B**	
P12: DMSO vs INS018_055	4.124
P15: DMSO vs INS018_055	3.879
P16: DMSO vs INS018_055	3.697
**IL6**	
P16: DMSO vs INS018_055	28.671
P18: DMSO vs INS018_055	7.367
**IL8**	
P16: DMSO vs INS018_055	5.637
P18: DMSO vs INS018_055	7.219
**TGFB1**	
P12: DMSO vs INS018_055	8.796

© 2024. Tang Q et al. Published online at http://www.aginganddisease.org/EN/10.14336/AD.2024.1492

P15: DMSO vs INS018_055	4.246
P16: DMSO vs INS018_055	7.590
P17: DMSO vs INS018_055	10.592
P18: DMSO vs INS018_055	11.394
**Supplementary Figure 2**	
**(A) SA-β-gal positive cell number**	
DMSO vs Doxo	16.428
Doxo vs ABT-263_1μM	2.449
**Total cell number**	
DMSO vs Doxo	10.464
Doxo vs ABT-263_1μM	2.449
**(B)**	
**SA-β-gal positive cell number**	
DMSO vs Doxo	13.559
Doxo vs RAPA_12.5nM	2.884
Doxo vs RAPA_25nM	3.269
Doxo vs RAPA_50nM	3.145
Doxo vs RAPA_100nM	2.361
**SA-β-gal positive%**	
DMSO vs Doxo	27.929
Doxo vs RAPA_12.5nM	3.155
Doxo vs RAPA_25nM	3.343
Doxo vs RAPA_50nM	2.597
Doxo vs RAPA_100nM	1.548
**(C)**	
**SA-β-gal positive cell number**	
DMSO vs Doxo	13.559
Doxo vs Torin 1_25nM	1.544
Doxo vs Torin 1_50nM	3.316
Doxo vs Torin 1_100nM	3.682
**SA-β-gal positive%**	
DMSO vs Doxo	27.929
Doxo vs Torin 1_50nM	3.485
Doxo vs Torin 1_100nM	3.238
**(E)Total cell number**	
DMSO vs RAPA-50nM	2.449
DMSO vs INS018_055_5μM	1.899
DMSO vs INS018_055 10μM	1.899
**Supplementary Figure 3**	
**(A) SA-β-gal positive cell number**	
DMSO vs RAPA-100nM	4.421
DMSO vs RAPA-50nM	5.234
DMSO vs ABT-263_1μM	1.791
DMSO vs INS018_055_1.25μM	2.078
DMSO vs INS018_055_2.5μM	2.078
DMSO vs INS018_055_5μM	2.078
DMSO vs INS018_055_10μM	2.078
**(B) SA-β-gal positive%**	
DMSO vs RAPA-100nM	3.715
DMSO vs RAPA-50nM	4.748
DMSO vs ABT-263_1μM	2.078
DMSO vs INS018_055_1.25μM	2.078
DMSO vs INS018_055_2.5μM	2.078
DMSO vs INS018_055_5μM	1.791
DMSO vs INS018_055_10μM	2.078
**(C)Total cell number**	
DMSO vs RAPA-100nM	3.018

© 2024. Tang Q et al. Published online at http://www.aginganddisease.org/EN/10.14336/AD.2024.1492

DMSO vs RAPA-50nM	3.579
DMSO vs ABT-263_1μM	2.078
DMSO vs INS018_055_2.5μM	2.078
DMSO vs INS018_055_5μM	2.078
DMSO vs INS018_055_10μM	2.078
**(D) SA-β-gal positive cell number**	
DMSO vs ABT-263_1μM	1.440
DMSO vs INS018_055_10μM	1.393
DMSO vs INS018_055_20μM	1.800
**(E) SA-β-gal positive%**	
DMSO vs INS018_055_5μM	1.206
DMSO vs INS018_055_10μM	1.538
DMSO vs INS018_055_20μM	1.884
**(F)Total cell number**	
DMSO vs ABT-263_1μM	1.458
**(G)TNIK**	
siNC vs si-TNIK-1	3.998
**(H) SA-β-gal positive cell number**	
siNC vs si-TNIK-1	1.525
siNC vs si-TNIK-2	5.838
siNC vs INS018_055_1.25μM	7.794
**(I) SA-β-gal positive%**	
siNC vs si-TNIK-1	1.322
siNC vs si-TNIK-2	3.929
siNC vs INS018_055_1.25μM	5.382
**Supplementary Figure 4**	
**(A) SA-β-gal positive%**	
P14 vs P15	2.582
P14 vs P16	2.828
P14 vs P17	2.828
P14 vs P18	2.828
P14 vs P19	2.928
P14 vs P20	2.828

Glossary of terms

**Terms**	**Explanation**
Senomorphics	therapeutic small molecules capable of suppressing senescent cell characteristics by blocking SASP
Cellular senescence	a cellular status characterized by stable exit from the cell cycle and loss of proliferative capacity even with growth-promoting stimuli
Senescence-associated secretory phenotype (SASP)	the secretory phenotype produced by senescent cells, including metalloproteinases, cytokines, chemokines, and growth factors, as well as non-protein metabolites
Senolytics	therapeutic small molecules that can kill senescent cells
Senostasis	approaches to reduce the detrimental impact of senescent cells by suppressing senescent traits
Artificial intelligence (AI)	a technical and scientific field devoted to the engineered system that generates outputs such as content, forecasts, recommendations, or decisions for a given set of human-defined objectives
Fibrosis	the process of replacing functional tissue with excess fibrous connective tissue under damage, leading to a reduction in organ function and ultimately organ failure and death
Geroprotectors	anti-aging interventions that can extend lifespan or health span
TGF-β signaling	transforming growth factor-β signaling that plays a critical role in the regulation of cell growth, differentiation, and development

© 2024. Tang Q et al. Published online at http://www.aginganddisease.org/EN/10.14336/AD.2024.1492

SUPPLEMENTARY DATA

c-Jun N-terminus kinase (JNK)	a family of protein kinases binding to and phosphorylate c-Jun that play a central role in stress signaling. The targets of JNK pathway include c-Jun, ATF2, ELK1, SMAD4, p53 *etc*
INS018_055	first AI-designed drug developed by Inisilico Medicine for IPF;
Idiopathic pulmonary fibrosis (IPF)	an aggressive interstitial lung disease with a high mortality rate
Extracellular matrix (ECM)	a large network of proteins and other molecules that surround, support, and give structure to cells and tissues in the body
Epithelial-to-mesenchymal transition (EMT)	a process by which epithelial cells lose their cell polarity and cell-cell adhesion, and gain migratory and invasive properties to become mesenchymal stem cells
Fibroblast-to-myofibroblast transition (FMT)	in the pathogenesis of fibrotic diseases or wound healing, a process that the fibroblasts at the quiescent state could be activated into the myofibroblast
Telomere attrition	a process that telomeres undergo shortening during cell division leading to cell senescence
Oxidative stress	an imbalance between the production and accumulation of oxygen-reactive species in cells and the ability of a biological system to remove these reactive products
High-content imaging	an image-based technology that can identify small molecules, peptides, or other substances that alter cellular phenotypes by extracting multiple cellular features such as morphology, localization, movements, *etc*. at a single cell level
Wnt-signaling	a pathway can regulate stem cell pluripotency and cell fate decisions during development. It can also interact with other singalongs such as TGF-β
Senescence-associated-β-galactosidase (SA-β-gal)	a lysosomal hydrolase with optimal activity at pH 6.0 in the senescent cells
ABT-263	also known as Navitoclax, a potent active Bcl-2 family protein inhibitor that binds to multiple anti-apoptotic Bcl-2 family proteins
Rapamycin	a potent and specific mTOR inhibitor
Torin 1	a potent inhibitor of mTOR
Replicative senescence	a process that normal somatic cells reach the irreversible cell cycle arrest following multiple rounds of replication

© 2024. Tang Q et al. Published online at http://www.aginganddisease.org/EN/10.14336/AD.2024.1492
